# Bioinspired Honokiol Analogs and Their Evaluation for Activity on the Norepinephrine Transporter

**DOI:** 10.3390/molecules23102536

**Published:** 2018-10-04

**Authors:** Kristen Stout, Marketa Bernaskova, Gary W. Miller, Antje Hufner, Wolfgang Schuehly

**Affiliations:** 1 Rollins School of Public Health, Emory University, 1518 Clifton Road, NE, Claudia Nance Rollins Bldg, Atlanta, GA 30322, USA; kristen.stout@gmail.com (K.S.); gwmille@emory.edu (G.W.M.); 2 Columbia University Mailman School of Public Health, 722 West 168th Street, Room 1411B, New York, NY 10032, USA; 3 Institute of Pharmaceutical Sciences, Pharmaceutical Chemistry, Universitätsplatz 1, University of Graz, 8010 Graz, Austria; mbernaskova@centrum.cz (M.B.); antje.huefner@uni-graz.at (A.H.); 4 Institute of Pharmaceutical Sciences, Pharmacognosy, Universitätsplatz 4, University of Graz, 8010 Graz, Austria; 5 Institute of Biology, Universitätsplatz 2, University of Graz, 8010 Graz, Austria

**Keywords:** honokiol derivatives, norepinephrine transporter, HEK cell expressing hNET, biphenyl-type neolignans, diphenylmethanes, *Magnolia officinalis*

## Abstract

In traditional Asian medicinal systems, preparations of the root and stem bark of *Magnolia* species are widely used to treat anxiety and other nervous disturbances. The biphenyl-type neolignans honokiol and magnolol are the main constituents of *Magnolia* bark extracts. In the central nervous system, *Magnolia* bark preparations that contain honokiol are thought to primarily interact with γ-aminobutyric acid A (GABA_A_) receptors. However, stress responses inherently involve the noradrenergic system, which has not been investigated in the pharmacological mechanism of honokiol. We present here interactions of honokiol and other synthesized biphenyl-type neolignans and diphenylmethane analogs with the norepinephrine transporter (NET), which is responsible for the synaptic clearance of norepinephrine and the target of many anxiolytics. Of the synthesized compounds, 16 are new chemical entities, which are fully characterized. The 52 compounds tested show mild, non-potent interactions with NET (IC_50_ > 100 µM). It is thus likely that the observed anxiolytic effects of, e.g., *Magnolia* preparations, are not due to direct interaction with the noradrenergic system.

## 1. Introduction

For centuries, traditional Chinese and Japanese medicinal preparations from *Magnolia* bark have been used to treat neurological diseases, including anxiety and sleep disorders [1]. Study of these preparations led to the identification of the biphenyl neolignans honokiol (**H**) and magnolol (**M**) as the major active constituents [2]. While these compounds act promiscuously in the periphery [3], the central nervous system activity of **H** and **M** has only been linked to interaction with GABA_A_ receptors [4].

It is possible that these biphenyl-type neolignans exert pharmacological activity on other CNS targets. In Western medicine, anxiolytic and antidepressant therapeutics (e.g., norepinephrine reuptake inhibitors, norepinephrine-dopamine reuptake inhibitors, or tricyclic antidepressants) generally increase noradrenaline levels by inhibiting the norepinephrine transporter (NET) [5]. NET localizes to the presynaptic membrane, where it acts to clear synaptic norepinephrine, thereby terminating signalling and recycling the neurotransmitter for subsequent release. Given the anxiolytic efficacy of **H** and **M**, we hypothesized biphenyl neolignans may similarly inhibit NET, a question that has not been addressed in the context of active ingredients from *Magnolia*-containing preparations. In addition to anxiolytic treatment, NET inhibitors are effective in the treatment of depression and attention deficit hyperactive disorder (ADHD). It is also considered a target for therapeutics against neurodegenerative diseases, such as Alzheimer’s and Parkinson’s disease [6], and in sleep regulation [7]. Thus, these compounds may offer therapeutic efficacy for a myriad of CNS disturbances.

**Figure 1:**
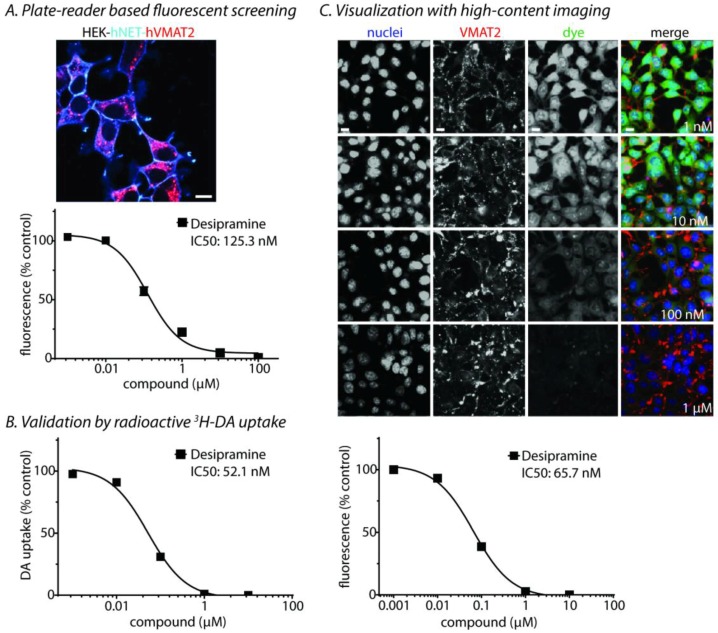
Plate reader-based fluorescent assay using human NET in a validation assay of the scree-ning strategy using the known NET inhibitor desipramine as a positive control. Compound screening workflow: (**A**) All compounds were screened for fluorescent uptake in human embryonic cidney HEK-hNET-hVMAT2 cells (blue hNET, red VMAT2) using a dye that is a substrate for NET, as indicated by inhibition by addition of a selective NET inhibitor, desipramine. (**B**) Next, fluorescent counts were confirmed with a radiolabelled uptake of dopamine, which is also a substrate of NET, as demonstrated by the lack of uptake in the presence of desipramine. (**C**) Finally, uptake was visualized using high content imaging with the Array Scan VTI HCS (Cellomics, Thermo Fisher Scientific, Waltham, MA, USA) to visualize nuclei (stained with DAPI), VMAT2-mCherry, and dye. All experiments were conducted in triplicate and analyzed with nonlinear regression analysis in GraphPad Prism (GraphPad Software, La Jolla, CA, USA). Scale: 10 nm.

Biphenyls are considered privileged structures due to their over-proportionally frequent biological activity [8]. In our previous investigations, we focused on the diverse biological activities of **H**, **M**, and derivatives thereof, such as COX-1/2, 5-LOX and LTB_4_-formation [9], GABA_A_ receptor activity [10,11], CB_2_ receptor activity [12], and toxicity on several cancer cell lines [13]. In this paper, we focus on the activity of the compounds at the NET. We tested 32 compounds of our already existing compound library and 16 additional compounds, of which 14 are new chemical substances (see Table 1, Table 2, Table 3, Table 4 and Table 5). Altogether 52 compounds have been evaluated in a norepinephrine (NE) uptake assay using a human embryonic kidney cell line stably expressing human NET (hNET) and human vesicular monoamine transporter 2 (VMAT2); human VMAT2 (hVMAT2) was added in addition to hNET to more faithfully recapitulate norepinephrine neurons in vitro (see characterization in Figure 1).

## 2. Results and Discussion

Previous studies indicated a significant increase in pharmacological activity when nitrogen-containing functional groups were introduced into **H** and other compounds [10]. We enhanced our existing compound library with further nitrogen-bearing honokiol derivatives with significantly changed polarity, either by *N*-acylation of 3-amino-4′-*O*-methylhonokiol (**H1**) with a long chain fatty acid (**H2**) or an amino acid (**H3**, **H4**), by formally replacing the methoxy group of **H1** with an ethoxy group (**H5**), or di-*O*-alkylation without or with *N*-acetylation (**H6**, **H7**). In contrast to amid **H8** of our parent library, in **H9** acetamido and *O*-methyl groups are located at C-5′ and C-2, respectively. In the course of the syntheses, intermediates were fully characterized by their chemical and physical properties, but not tested in the biological assay. Compound data is provided for all synthesized chemicals, including compounds mentioned in the literature for which chemical and physical data were not fully provided.

All synthesized compounds were tested for pharmacological activity at the NET and compared with selective NET inhibitor, desipramine (Figure 1). Compounds with any apparent pharmacological activity were further characterized by radioactive uptake and high content imaging. No compound showed robust NET activity (Table 1).

**Scheme 1:**
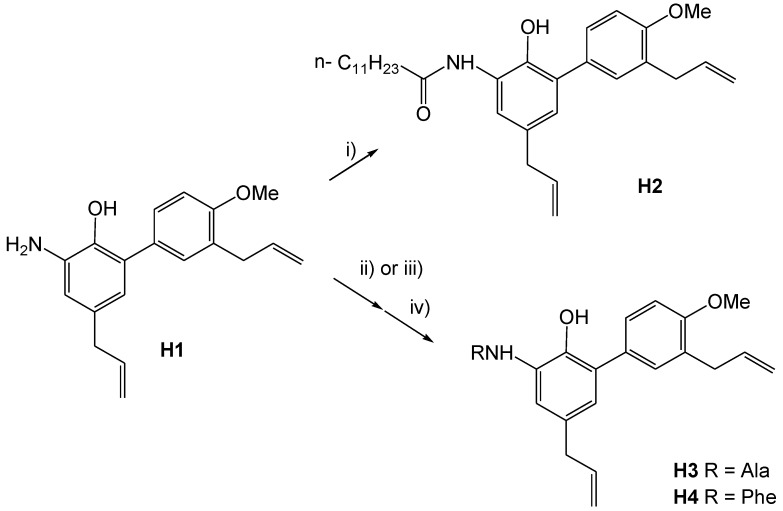
Synthesis of honokiol derivatives. Reagents and conditions: i) *n*-C_11_H_23_COCl, pyridine; ii) Fmoc-l-Ala-Cl, Zn, THF; iii) Fmoc-l-Phe-Cl, Zn, THF; iv) piperidine.

**Scheme 2:**
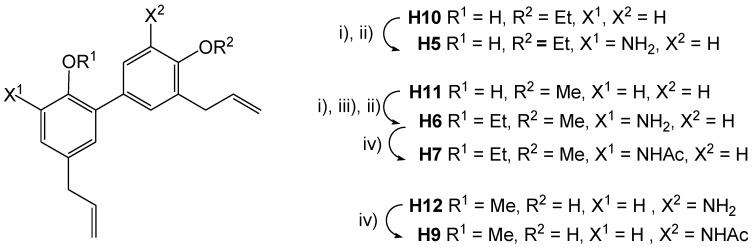
Synthesis of honokiol derivatives. Reagents and conditions: i) HNO_3_ (65%), AcOEt; ii) SnCl_2_×2 H_2_O, EtOH; iii) MW irradiation 1. KOH, 2. Me_2_SO_4_ or Et_2_SO_4_; iv) Ac_2_O, H_2_O.

As outlined in Scheme 1, *N*-acylations of **H1** were performed using the corresponding carboxylic acid chlorides [14,15]. An Fmoc-protection group was used for the preparation of the peptide-type amides **H3** and **H4** [16,17] (see Scheme 1). *N*-acylation of **H6** resulted in **H7** (see Scheme 2).

As outlined in Scheme 2, **H5** was prepared analogously to **H1** from 4′-*O*-ethylhonokiol (**H10**) via nitration with nitric acid followed by reduction with SnCl_2_ × 2H_2_O [10,18,19]. To avoid *N*-alkylation in the synthesis of di-*O*-alkylated amine **H6**, the *O*-alkylation of the hydroxy group of 4′-*O*-methylhonokiol honokiol (**H11**) had to be performed after nitration and prior to reduction with SnCl_2_ × 2 H_2_O. *N*-acetylation of **H6** with acetic anhydride resulted in **H7 [10]**. **H9** was prepared by a similar *N*-acetylation of the corresponding amine **H12**.

**Scheme 3:**
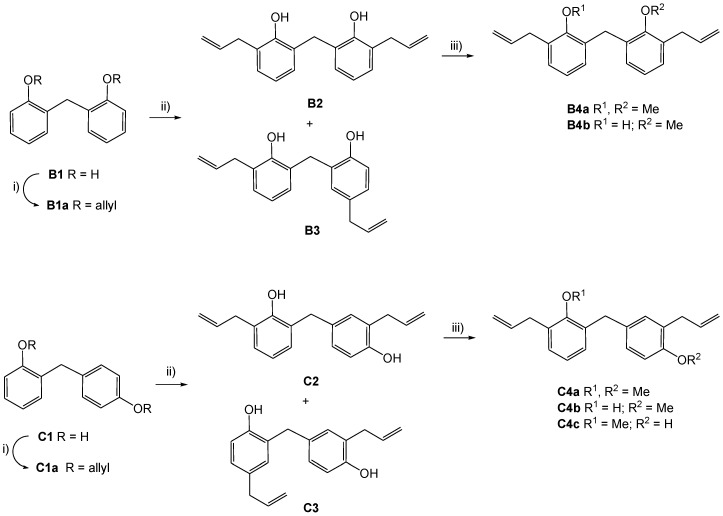
Synthesis of diphenylmethane derivatives. Reagents and conditions: i) K_2_CO_3_, CH_2_=CH–CH_2_–Br, acetone; ii) Δ, Ph-NMe_2_; iii) 1. KOH, 2. Me_2_SO_4_.

To go one step further, we tested several dioxygenated diphenylmethanes to see if insertion of a methylene group between the two aromatic moieties affects biological activity. As outlined in Scheme 3, the synthesis started from bis(2-hydroxyphenyl)methane (**B1**) and 2,4′-dihydroxydiphenylmethane (**B1**), which are commercially available but were also prepared from salicylic alcohol and phenol [20]. Double *O*-allylation and Claisen rearrangement according to Chattopadhyay et al. [21] yielded mainly the *ortho* rearrangement products (**B2** and **C2** resp.) together with little of the *para* rearrangement products (**B3** and **C3** resp.). **B2** and **C2** were submitted to incomplete *O*-methylation to yield the monoethers **B4a**, **C4b**, and **C4c** together with the respective diether **B4a** or **C4a**.

## 3. Experimental

### 3.1. General

Microwave reactions were carried out on a CEM Corp. Discover laboratory microwave equipped with an Explorer unit (CEM Corp. Matthews, NC, USA). Infrared spectra were recorded on a Bruker Alpha Platinum ATR spectrometer (Bruker, Kennewick, WA, USA) or as KBr pellets on a Perkin-Elmer 281 B spectrometer (PerkinElmer, Waltham, MA, USA). ^1^H and ^13^C-NMR spectra were recorded on a Varian 400 MHz UnityINOVA spectrometer (400 and 100 MHz, respectively, Varian, Palo Alto, CA, USA) and unless otherwise stated in CDCl_3_ with undeuterated solvent as an internal standard (7.26 ppm and 77.0 ppm, respectively). For convenience, atoms were numbered according to structures in Table 1, Table 4 and Table 5 with double-primed numbers for substituents at ring A and triple primed numbers for substituents at ring B, respectively. ESI-MS were recorded in ESI positive and negative mode on an LC Ultimate 3000 (Thermo, San José, CA, USA) with DAD detection in line with a Thermo Scientific LTQ XL mass spectrometer. Column: Knauer (Berlin, Germany) RP-18 (1.8 μm; 125 × 2.1 mm) with a guard cartridge at a flowrate of 150 μL/min. ESI-MS were recorded on an Agilent Technologies HP 7890A instrument fitted with detector HP 5975C VL MSD (70 eV, ion source 250 °C, quadrupole temperature 150 °C, Santa Clara, CA, USA). An Agilent HP-5MS column (30 m, ID 0.25 mm, film 5%pheny l95%, methylpolysiloxane 0.25 μm) was used. The oven temperature was kept at 45 °C for 2 min and programmed to increase to 300 °C at a rate of 3 °C/min, then kept constant at 300 °C for 20 min, with a total run time of 64.5 min. Helium was used as a carrier gas. The injection volume was 1 μL (≈0.5% solution) and a split ratio of 1:50.

For high resolution mass spectrometry, a Waters GCT Premier instrument was used with electron impact ionization (70 eV) at an ion source temperature of 200 °C.

#### General Information on Syntheses

Compounds were synthesized as described below. Solvents were of analytical quality, if not stated otherwise. The purity of all synthesized compounds was verified using NMR and analytical HPLC. Analytical thin layer chromatography (TLC) was performed using aluminium foil coated with silica 60 F_254_ (Merck, Darmstadt, Germany). Preparative thin layer chromatography (PTLC) was performed using glass plates coated with silica 60 F_254_ (Merck). Detection was done using UV/254 nm and spraying with molybdophosphoric acid and subsequent heating. Compound mixtures were separated using column chromatography (CC) on silica gel 60 (63–200 µm, Merck) using cyclohexane/ethyl acetate mixtures. Further purification was performed using preparative HPLC (Varian Prepstar with a Dynamax Rainin detector; column SepServ, Berlin, Germany, 250 × 21 mm, RP-18, 7 μm, flow rate 15 mL). Honokiol (purity > 98%) was purchased from APIChem Technology Co. (Hangzhou, China). Proton NMR spectra of the newly described compounds are given under “Supplementary Materials”.

3-Dodecanoylamino-4′-*O*-methylhonokiol (**H2**)

Dodecanoyl chloride (0.34 mmol, 80 μL) was added with intense stirring at room temperature (RT) to a solution of 100 mg (0.34 mmol) of **H1** and pyridine (0.41 mmol, 33 μL) in abs. Et_2_O (4 mL). The reaction mixture was stirred at room temperature overnight and filtered. The precipitate was extracted with Et_2_O (4 mL). NaHCO_3_ (1 M, 10 mL) was added to the combined filtrate and washings. The organic phase was separated, and the aqueous phase was extracted with Et_2_O (3 × 10 mL). The combined organic phases were washed with brine (10 mL), dried over Na_2_SO_4_, concentrated under reduced pressure, and purified using CC (silica, cyclohexane/AcOEt 5:1) resulting in 107 mg (59%) of a light brown solid (**H2**).

Compound **H2**: IR (ATR, *ν*_max_, cm^−1^): 3304, 2956, 2919, 2847, 2485, 1639, 1606, 1539, 1499, 1467, 1411, 1243, 906, 725, 577; ^1^H-NMR (CDCl_3_) δ 7.57 (bs, 1H, NH), 7.40 (d, *J* = 1.5 Hz, 1H, H-4), 7.34 (dd, *J* = 8.4, 2.3 Hz, 1H, H-6′), 7.27 (d, *J* = 2.1 Hz, 1H, H-2′), 7.17 (s, 1H, OH), 6.94 (d, *J* = 8.4 Hz, 1H, H-5′), 6.89 (d, *J* = 1.9 Hz, 1H, H-6), 6.01 (ddt, *J* = 16.8, 10.2, 6.6 Hz, 1H, H-2‴), 5.96 (ddt, *J* = 16.8, 10.0, 6.7 Hz, 1H, H-2″), 5.02–5.13 (m, 4H, H-3‴, H-3″), 3.87 (s, 3H, OCH_3_), 3.43 (d, *J* = 6.6 Hz, 2H, H-1‴), 3.33 (d, *J* = 6.7 Hz, 2H, H-1″), 2.42 (t, *J* = 7.5 Hz, 2H, Acyl-H_α_), 1.74 (quint, *J* = 7.3 Hz, 2H, Acyl-H_β_), 1.21–1.42 (m, 16H, Acyl-H_γ_ to H_ω-1_), 0.88 (t, *J* = 6.8 Hz, 3H, Acyl-H_ω_), ^13^C-NMR (CDCl_3_) δ 172.7 (CO), 156.8 (C-4′), 142.3 (C-2), 137.5 (C-2″), 136.7 (C-2‴), 132.2 (C-5), 130.7 (C-2′), 130.7 (C-1), 129.6 (C-1′), 129.0 (C-3′), 128.1 (C-6′), 126.7 (C-6), 126.2 (C-3), 120.3 (C-4), 115.8 (C-3″), 115.6 (C-3‴), 110.5 (C-5′), 55.5 (OCH_3_), 39.5 (C-1″), 37.5 (Acyl-C_α_), 34.3 (C-1‴), 31.9 (Acyl-C_ω-2_), {2 × 29.6, 29.4, 2 × 29.3, 29.2 (Acyl-C_γ_-to C_ω-3_)}, 25.7 (Acyl-C_β_), 22.7 (Acyl-C_ω-1_), 14.1 (Acyl-C_ω_), ESI^+^ calcd for C_31_H_43_NO_3_: [M]^+^ 477.324; found ESI-MS *m*/*z* (rel. int.): 478.63 [M + H]^+^ (100). From the same batch, the compound has been tested in parallel towards CB_1_/CB_2_ receptor agonistic activity by Bertini et al. [22]; however, spectroscopic data and synthesis are only given here.

3-(*N*-l-Alanyl)-4′-*O*-methylhonokiol (**H3**)

l-*N*-(9-Fluorenylmethoxycarbonyl)alanylchloride, Fmoc-l-Ala-Cl: Fmoc-l-Ala-OH · H_2_O was first dehydrated at 50 °C over P_2_O_5_ in vacuo for 12 h. Under dry conditions 400 mg (1.28 mmol) l-*N*-(9-Fluorenylmethoxycarbonyl)alanin was suspended in 5 mL dichloromethane, thionyl chloride (freshly distilled, 1.23 mL, 17.7 mmol) was added, and the mixture was sonicated at RT. After 7 min, the reaction mixture was a homogeneous solution and it was sonicated for an additional 15 min. Dichloromethane and the excess of thionyl chloride were removed in vacuo to yield 404 mg (96%) of Fmoc-l-Ala-Cl as a grey solid, which was used without further purification for a second step.

l-3-(*N*-(9-Fluorenylmethoxycarbonyl)alanyl)-4′-*O*-methylhonokiol (**H3a**): Zn (dust) was treated with HCl (2 M, aqueous solution), washed subsequently with water, EtOH and diethylether, dried in vacuo, and stored under argon. Under argon, **H1** (259 mg, 0.841 mmol), Fmoc-l-Ala-Cl (279 mg, 0.846 mmol), and Zn dust (55 mg, 0.841 mmol) were suspended in abs. THF (50 mL) and stirred at RT overnight. Over a period of two hours, the reaction color turned from brownish to yellowish. Reaction completion was proven using TLC analysis (cyclohexane/AcOEt = 5:3). Zn dust was filtered off and the filtrate was evaporated in vacuo*.* The residue was dissolved in dichloromethane (15 mL), washed with brine (2 × 5 mL), and dried over Na_2_SO_4_. Evaporation of the solvent in vacuo and CC (silica, cyclohexane/AcOEt = 5:1) yielded in **H3a** (428 mg; 87%).

3-(*N*-l-Alanyl)-4′-*O*-methylhonokiol (**H3**): **H3a** (401 mg, 0.682 mmol) was dissolved in diethyl ether (3 mL) and piperidine (1 mL, 1 mmol) was added. The mixture was stirred at RT for 1 h. The reaction mixture was evaporated under reduced pressure and dichloromethane (5 mL) was added to the residue. The resulting precipitate was filtered off and the filtrate was concentrated under reduced pressure to dryness. The residue (377 mg) was purified using CC (silica, dichloromethane/MeOH, gradient: 0–10% *v*/*v* methanol) to yield 220 mg (88%) of **H3** as an orange oil.

Compound **H3**: IR (ATR, *ν*_max_, cm^−1^): 3272, 3075, 2974, 2929, 2834, 1638, 1606, 1590, 1530, 1500, 1460, 1440, 1244, 1029, 993, 910, 813, 594; ^1^H-NMR (CDCl_3_ + D_2_O) δ 7.35 (dd, *J* = 8.1, 1.8 Hz, 1H, H-6′), 7.29 (d, *J* = 2.2 Hz, 1H, H-2′), 7.18 (s, 1H, H-4), 6.92 (s, 1H, H-6), 6.91 (d, *J* = 8.4 Hz, 1H, H-5′), 6.01 (ddt, *J* = 16.8, 9.8, 6.6 Hz, 1H, H-2‴), 5.95 (ddt, *J* = 16.9, 9.9, 6.6 Hz, 1H, H-2″), 5.01–5.13 (m, 4H, H-3″, H-3‴), 3.85 (s, 3H, O–CH_3_), 3.73 (q, *J* = 6.2 Hz, 1H, CH_3_C*H*(NH_2_)CO), 3.42 (d, *J* = 6.6 Hz, 2H, H-1‴), 3.32 (d, *J* = 6.2 Hz, 2H, H-1″), 2.03 (s, 2H, N*H*_2_), 1.45 (d, *J* = 6.6 Hz, 3H, C*H*_3_CH(NH_2_)CO); ^13^C-NMR (CDCl_3_ + D_2_O) δ 174.6 (CO), 156.7 (C-4′), 143.2 (C-2), 137.5 (C-2″), 136.8 (C-2‴), 131.9 (C-5), 131.0 (C-1), 130.8 (C-2′), 130.1 (C-1′), 128.6 (C-3′), 128.2 (C-6′), 127.5 (C-6), 125.6 (C-3), 120.6 (C-4), 115.7 (C-3″), 115.5 (C-3‴), 110.3 (C-5′), 55.5 (O–CH_3_), 50.5 (CH_3_*C*H(NH_2_)CO), 39.4 (C-1″), 34.3 (C-1‴), 21.1 (*C*H_3_CH(NH_2_)CO); ESI^+^ calcd for C_22_H_26_N_2_O_3_: [M]^+^ 366.194; found ESI-MS *m*/*z* (rel. int.): 367.18 [M + H]^+^ (100).

3-(*N*-l-Phenylalanyl)-4′-*O*-methylhonokiol (**H4**)

l-*N*-(9-Fluorenylmethoxycarbonyl)phenylalanylchloride, Fmoc-l-Phe-Cl: Under dry conditions 300 mg (0.774 mmol) l-*N*-(9-Fluorenylmethoxycarbonyl)phenylalanin was suspended in dry dichloromethane (4 mL), thionyl chloride (freshly distilled, 0.776 mL, 10.7 mmol) was added, and the mixture was sonicated at RT for 45 min. The sonication bath was heated up to 40 °C over that period of time. Dichloromethane and the excess of thionyl chloride were removed in vacuo to yield 294 mg (94%) of Fmoc-l-Phe-Cl as a grey solid, which was used without further purification in the next step.

3-(*N*-l-Phenylalanyl)-4′-*O*-methylhonokiol (**H4**): Zn dust was treated with aqueous HCl (2 M), washed subsequently with water, ethanol, and diethylether, dried in vacuo, and stored under argon. Under argon, **H1** (75 mg, 0.25 mmol), Fmoc-l-Phe-Cl (103 mg, 0.254 mmol), and Zn dust (17 mg, 0.25 mmol) were suspended in dry THF (10 mL) and stirred for 4 h. Reaction completion was proven using TLC analysis (cyclohexane/AcOEt = 5:3). Piperidine (0.5 mL, 5 mmol) was added and the reaction mixture was stirred for 20 min at RT, the solvent was evaporated under reduced pressure and the residue was diluted with dichloromethane (5 mL), filtered, and evaporated under reduced pressure. The residue (170 mg) was purified using CC (silica, cyclohexane/AcOEt, gradient: 0–100% *v*/*v* AcOEt) to yield 50 mg (45%) of **H4** as an orange oil.

Compound **H4**: IR (ATR, *ν*_max_, cm^−1^): 3270, 3073, 2974, 2834, 1638, 1605, 1530, 1500, 1453, 1439 s, 1244, 1028, 910; ^1^H-NMR (CDCl_3_) δ 9.69 (bs, 1H, CON*H*), 7.39 (dd, *J* = 8.4, ≈2 Hz, 1H, H-6′), 7.30–7.36 (m, 3H, Ph-H_meta_ Ph-H_para_), 7.22–7.30 (m, 3H, H-2′, Ph-H_ortho_), 7.10 (s, 1H, H-4), 6.95 (s, 1H, H-6), 6.92 (d, *J* = 8.4 Hz, 1H, H-5′), 6.01 (ddt, *J* = 16.9, 9.9, 6.6 Hz, 1H, H-2‴), 5.97 (ddt, *J* = 16.9, 10.2, 6.6 Hz, 1H, H-2″), 5.02–5.14 (m, 4H, H-3″, H-3‴), 3.86 (s, 3H, OCH_3_), 3.82 (m, 1H, PhCH_2_C*H*(NH_2_)CO), 3.43 (d, *J* = 6.6 Hz, 2H, H-1‴), 3.34 (m, 1H, PhC*H*_2_CH(NH_2_)CO), 3.33 (d, *J* = 6.9 Hz, 2H, H-1″), 2.84 (dd, *J* = 13.6, 9.2 Hz, 1H, PhC*H*_2_CH(NH_2_)CO); ^13^C-NMR (CDCl_3_) δ 173.7 (CO), 156.6 (C-4′), 143.5 (C-2), 139.1 (Ph-C_ipso_), 137.5 (C-2″), 136.8 (C-2‴), 131.8 (C-5), 131.2 (C-1), 130.8 (C-2′), 130.2 (C-1′), 129.3 (Ph-C_ortho_), 128.8 (Ph-C_meta_), 128.6 (C-3′), 128.2 (C-6′), 127.6 (C-6), 127.0 (Ph-C_para_), 125.7 (C-3), 120.5 (C-4), 115.7 (C-3″), 115.5 (C-3‴), 110.3 (C-5′), 56.3 (PhCH_2_*C*H(NH_2_)CO), 55.5 (OCH_3_), 40.5 (Ph*C*H_2_CH(NH_2_)CO), 39.3 (C-1″), 34.3 (C-1‴); ESI^+^ calcd for C_28_H_30_N_2_O_3_: [M]^+^ 442.226; found ESI-MS *m*/*z* (rel. int.): 442.20 [M + H]^+^ (100).

3-Amino-4′-*O*-ethylhonokiol (**H5**)

4′-*O*-Ethyl-3-nitro-honokiol (**H5a**): Aqueous nitric acid (65%, 0.474 mL, 6.79 mmol) was added under intense stirring within ≈5 s to a solution of **H10** (200 mg, 0.679 mmol; synthesis see Schuehly et al. [12] supplemental; cpd **16b**) in AcOEt (5 mL) at RT. The reaction mixture was stirred for 60 s and quenched with NaHCO_3_ (1 M, 10 mL). The organic phase was separated, and the aqueous phase was extracted with AcOEt (3 × 10 mL). The combined organic layers were washed with brine (10 mL), dried over Na_2_SO_4_, concentrated under reduced pressure, and purified using CC (silica, cyclohexane/AcOEt 99:1) to yield 120 mg (50%) of 4′-*O*-ethyl-3-nitro-honokiol (**H5a**) as a dark yellow oil.

Compound **H5a**: IR (ATR, *ν*_max_, cm^−1^): 3177, 3078, 2978, 2903, 1638, 1607, 1537, 1502, 1314, 1244, 1045, 912, 672, 555 cm^−1^; ^1^H-NMR (CDCl_3_) δ 11.03 (s, 1H, O*H*), 7.90 (d, *J* = 2.2 Hz, 1H, H-4), 7.45 (d, *J* = 2.2 Hz, 1H, H-6), 7.38 (dd, *J* = 8.4, 2.2, 1H, H-6′), 7.33 (d, *J* = 2.2 Hz, 1H, H-2′), 6.92 (d, *J* = 8.4 Hz, 1H, H-5′), 6.03 (ddt, *J* = 16.9, 10.3, 6.6 Hz, 1H, H-2‴), 5.96 (ddt, *J* = 16.5, 10.3, 6.6 Hz, 1H, H-2″), 5.03–5.17 (m, 4H, H-3″ and H-3‴), 4.10 (q, *J* = 7.2 Hz, 2H, OC*H*_2_CH_3_), 3.45 (d, *J* = 6.4 Hz, 2H, H-1‴), 3.40 (d, *J* = 6.6 Hz, 2H, H-1″), 1.46 (t, *J* = 6.9 Hz, 3H, OCH_2_C*H*_3_); ^13^C-NMR (CDCl_3_) δ 156.6 (C-4′), 151.3 (C-2), 138.9 (C-6), 136.8 (C-2‴), 136.0 (C-2″), 133.8 (C-3), 132.9 (C-1), 131.7 (C-5), 130.7 (C-2′), 128.5 (C-3′), 128.2 (C-6′), 127.4 (C-1′), 122.8 (C-4), 117.1 (C-3‴), 115.6 (C-3″), 111.0 (C-5′), 63.7 (O*C*H_2_CH_3_), 38.9 (C-1″), 34.5 (C-1‴), 14.9 (OCH_2_*C*H_3_); ESI^–^ calcd for C_20_H_21_NO_4_: [M]^−^ 339.147; found ESI-MS *m*/*z* (rel. int.): 338.21 ([M − H]^−^ (100).

3-Amino-4′-*O*-ethylhonokiol (**H5**): SnCl_2_×2H_2_O (1.62 g, 7.16 mmol) was added to a solution of **H5a** (270 mg, 0.796 mmol) in abs. MeOH (10 mL). The reaction mixture was stirred for 48 h at RT, concentrated under reduced pressure and diluted with AcOEt (15 mL). The spumy precipitate resulting from the addition of aqueous NaHCO_3_ (1 M, 20 mL) was filtered off with Celite^®^ (Sigma-Aldrich, Buchs, Switzerland) and rinsed with AcOEt (30 mL). The organic layer was separated from the combined filtrate and washings, dried over Na_2_SO_4_, and concentrated under reduced pressure. The residue (127 mg) was purified using PTLC (silica, cyclohexane/AcOEt = 5:1) to yield 65 mg (26%) of **H5** as a brown oil.

Compound **H5**: IR (ATR, *ν*_max_, cm^−1^): 3544, 3370, 3075, 2977, 2900, 1637, 1607, 1503, 1486,1474, 1242, 1215, 1122, 1044, 908, 808; ^1^H-NMR (CDCl_3_) δ 7.26 (d ≈ 9 Hz, 1H, H-6′), 7.25 (s, 1H, H-2′), 6.93 (d, *J* = 8.1 Hz, 1H, H-5′), 6.61 (s, 1H, H-4), 6.51 (s, 1H, H-6), 6.00 (ddt, *J* = 16.9, 9.9, 6.6 Hz, 1H, H-2‴), 5.96 (ddt, *J* = 17.0, 9.9, 6.9 Hz, 1H, H-2″), 5.02–5.14 (m, 4H, H-3″, H-3‴), 4.19 (bs, 2H, NH_2_), 4.09 (q, *J* = 7.0 Hz, 2H, OC*H_2_*CH_3_), 3.43 (d, *J* = 6.6 Hz, 2H, H-1‴), 3.28 (d, *J* = 6.9 Hz, 2H, H-1″), 1.46 (t, *J* = 7.0 Hz, 3H, OCH_2_C*H_3_*); ^13^C-NMR (CDCl_3_) δ 156.4 (C-4′), 139.0 (C-2), 137.9 (C-2″), 136.6 (C-2‴), 133.6 (C-3), 132.4 (C-5), 130.3 (C-2′), 129.9 (C-3′), 129.0 (C-1′), 127.9 (C-1), 127.7 (C-6′), 120.5 (C-6), 115.8 (C-3‴), 115.7 (C-4), 115.4 (C-3″), 111.9 (C-5′), 63.7 (O*C*H_2_CH_3_), 39.6 (C-1″), 34.5 (C-1‴), 14.9 (OCH_2_*C*H_3_); ESI^+^ calcd C_20_H_23_NO_2_: [M]^+^ 309.173; found ESI-MS *m*/*z* (rel. int.): 310.11 [M + H]^+^ (100).

3-Amino-2-*O*-ethyl-4′-*O*-methylhonokiol (**H6**)

In a microwave process vial, KOH (132 mg, 2.36 mmol) was added to a solution of 4′-*O*-methyl-3-nitro-honokiol (synthesis see [10]) (192 mg, 0.590 mmol) in abs. MeOH (2 mL) and the mixture was stirred at RT for 10 min. Diethyl sulfate (155 μL, 2.36 mmol) was added and the reaction mixture was irradiated in a microwave oven at 90 °C for 60 min. After cooling to RT, the reaction mixture was neutralized with aqueous HCl (2 M). MeOH was evaporated under reduced pressure (40 °C, 100 mbar). The residue was extracted with dichloromethane (3 × 2 mL), the organic phase washed with water (3 × 5 mL), dried over Na_2_SO_4_, and concentrated under reduced pressure, resulting in 198 mg of crude 2-*O*-ethyl-4′-*O*-methyl-3-nitro-honokiol, which was used without further purification.

SnCl_2_×2H_2_O (1.09 g, 4.84 mmol) was added to a solution of crude 2-*O*-ethyl-4′-*O*-methyl-3-nitro-honokiol (190 mg, 0.54 mmol) in abs. EtOH (10 mL). The reaction mixture was stirred at RT for 48 h. The spumy precipitate resulting from the addition of aqueous NaHCO_3_ (1 M, 20 mL) was filtered using Celite^®^, the residue was extracted with EtOH (50 mL). Combined organic layers were evaporated under reduced pressure (100 mbar). The residue was extracted with dichloromethane (3 × 10 mL), which was washed with brine (3 × 5 mL), dried over Na_2_SO_4_, and concentrated under reduced pressure to yield 107 mg of a crude product, which after purification using PTLC (silica, cyclohexane/AcOEt = 5:1), yielded 26 mg (14%) of **H6**.

Compound **H6**: IR (ATR, *ν*_max_, cm^−1^): 3417, 3310, 3076, 2975, 2928, 2836, 1674, 1638, 1607, 1522, 1502, 1435, 1245, 1026, 910, 812, 600, 523; ^1^H-NMR (CDCl_3_) δ 7.47 (d, *J* = 2.2 Hz, 1H, H-2′), 7.46 (dd, *J* = 8.1, 2.2 Hz, 1H, H-6′), 6.92 (d, *J* = 8.4 Hz, 1H, H-5′), 6.60 (s, 2H, H-4, H-6), 6.07 (ddt, *J* = 16.6, 10.2, 6.2 Hz, 1H, H-2‴), 6.00 (ddt, *J* = 16.9, 9.9, 6.9 Hz, 1H, H-2″), 5.04–5.17 (m, 4H, H-3″, H-3‴), 3.96 (bs, 2H, NH_2_), 3.89 (s, 3H, OCH_3_), 3.57 (q, *J* = 6.9 Hz, 2H, OC*H*_2_CH_3_), 3.46 (d, *J* = 6.6 Hz, 2H, H-1‴), 3.33 (d, *J* = 6.9 Hz, 2H, H-1″), 1.16 (t, *J* = 7.1 Hz, 3H, OCH_2_C*H*_3_); ^13^C-NMR (CDCl_3_) δ 156.4 (C-4′), 142.1 (C-2), 140.0 (C-3), 137.6 (C-2″),137.0 (C-2‴), 136.0 (C-5), 134.4 (C-1), 131.0 (C-1′), 130.5 (C-2′), 128.0 (C-3′), 127.6 (C-6′), 120.5 (C-6), 115.5 (C-3″), 115.2 (C-3‴), 114.7 (C-4), 109.9 (C-5′), 67.6 (C–O*C*H_2_CH_3_), 55.4 (OCH_3_), 39.9 (C-1″), 34.3 (C-1‴), 15.7 (OCH_2_*C*H_3_); ESI^+^ calcd for C_21_H_25_NO_2_: [M]^+^ 323.189; found ESI-MS *m*/*z* (rel. int.): 324.23 [M + H]^+^ (100).

3-Acetylamino-2-*O*-ethyl-4′-*O*-methylhonokiol (**H7**)

In a 10 mL round-bottom flask, **H6** (34 mg, 0.105 mmol) was mixed with water (0.3 mL), and acetic anhydride (0.21 mmol, 20 μL) was added. The flask was rotated at 80 °C for about 10 min in a water bath. After cooling to RT, the reaction mixture was quenched with aqueous NaHCO_3_ (1 M, 2 mL) and extracted with dichloromethane (3 × 2 mL). The combined extracts were washed with aqueous NaHCO_3_ (1 M, 2 mL), water (2 mL), dried over Na_2_SO_4_, and concentrated under reduced pressure. The crude product (27 mg) was purified using PTLC (cyclohexane/AcOEt = 5:3), yielding 21 mg (55%) of **H7** as a brownish oil.

Compound **H7**: IR (ATR, *ν*_max_, cm^−1^): 3469, 3368, 3075, 2974, 2928, 2903, 2834, 1638, 1606, 1504, 1438, 1243, 1209, 1030, 909, 810, 596; ^1^H-NMR (CDCl_3_) δ 8.17 (s, 1H, H-4), 7.96 (bs, 1H, NH), 7.38 (s, 1H, H-2′), 7.37 (dd, *J* ≈ 8, 2.2 Hz, 1H, H-6′), 6.90 (d, *J* = 8.1 Hz, 1H, H-5′), 6.86 (d, *J* = 1.1 Hz, 1H, H-6), 6.03 (ddt, *J* = 16.8, 9.9, 6.9 Hz, 1H, H-2‴), 5.98 (ddt, *J* = 16.9, 9.9, 6.6 Hz, 1H, H-2″), 5.02–5.15 (m, 4H, H-3″, H-3‴), 3.87 (s, 3H, OCH_3_), 3.52 (q, *J* = 7.1 Hz, 2H, OC*H*_2_CH_3_), 3.42 (d, *J* = 6.6 Hz, 2H, H-1‴), 3.38 (d, *J* = 7.0 Hz, 2H, H-1″), 2.21 (s, 3H, CH_3_CO), 1.14 (t, *J* = 7.0 Hz, 3H, OCH_2_C*H*_3_); ^13^C-NMR (CDCl_3_) δ 168.09 (CO), 156.7 (C-4′), 143.1 (C-2), 137.3 (C-2″), 136.9 (C-2‴), 136.2 (C-5), 133.5 (C-1), 131.9 (C-3), 130.4 (C-1′), 130.3 (C-2′), 128.4 (C-3′), 127.6 (C-6′), 125.3 (C-6), 118.7 (C-4), 115.8 (C-3″), 115.4 (C-3‴), 110.2 (C-5′), 68.7 (O–*CH_2_*–CH_3_), 55.4 (OCH_3_), 40.0 (C-1″), 34.3 (C-1‴), 24.9 (*CH_3_*–C=O), 15.6 (OCH_2_*C*H_3_); ESI^+^ calcd for C_23_H_27_NO_3_: [M]^+^ 365.199; found ESI-MS *m*/*z* (rel. int.): 366.29 [M + H]^+^ (100).

5′-(*N*-Acetylamino)-2-*O*-methyl-honokiol (**H9**)

In a 10 mL round-bottom flask, **H12** (for synthesis, see Reference [11]) (37 mg, 0.125 mmol) was suspended with water (1 mL), and acetic anhydride (0.21 mmol, 20 μL) was added. The flask was allowed to rotate for 10 min at 80 °C in a water bath. After cooling to RT, the reaction mixture was quenched with aqueous NaHCO_3_ (1 M, 1 mL) and extracted with dichloromethane (3 × 1.5 mL). The combined extracts were washed with aqueous NaHCO_3_ (1 M, 1.5 mL) and water (1.5 mL), dried over Na_2_SO_4_, and concentrated under reduced pressure. The crude product (29 mg) was purified using PTLC (silica, cyclohexane/AcOEt 5:3), yielding 16 mg (39%) of **H9**, a light orange solid.

Compound **H9**: IR (ATR, *ν*_max_, cm^−1^): 3276, 3075, 3001, 2917, 2848, 1750, 1637, 1595, 1540, 1480, 1239, 1239, 1139, 1025, 910, 873, 809; ^1^H-NMR (CDCl_3_) δ 7.62 (s, br 1H, N*H*), 7.18 (s, 1H, H-2′), 7.10 (d ≈ 8 Hz, 1H, H-4), 7.07 (s, 2H, H-6, H-6′), 6.89 (d, *J* = 8 Hz, 1H, H-3), 6.06 (ddt, *J* = 16.9, 10.3, 6.6 Hz, 1H, H-2‴), 5.97 (ddt, *J* ≈ 17, 9.9, 6.9 Hz, 1H, H-2″), 5.03–5.16 (m, 4H, H-3″, H-3‴), 3.77 (s, 3H, OC*H*_3_), 3.50 (d, *J* = 5.9 Hz, 2H, H-1‴), 3.35 (d, *J* = 6.6 Hz, 2H, H-1″), 2.22 (s, 3H, CO-C*H*_3_); ^13^C-NMR (CDCl_3_) δ 170.5 (CO), 154.7 (C-2), 146.0 (C-4′), 137.7 (C-2″), 136.8 (C-2‴), 132.3 (C-5), 130.9 (C-6), 130.4 (C-5′), 130.2 (C-3′), 129.6 (C-1), 128.8 (C-2′), 128.2 (C-4), 125.2 (C-1′), 121.4 (C-6′), 115.7 (C-3‴), 115.6 (C-3″), 111.4 (C-3), 55.8 (O*C*H_3_), 39.4 (C-1″), 35.9 (C-1‴), 23.7 (CO–*C*H_3_); ESI^+^ calcd for C_21_H_23_NO_3_: [M]^+^ 337.168; found ESI-MS *m*/*z* (rel. int.): 338.08 [M + H]^+^ (100).

Bis(2-Allyloxyphenyl)-methane (**B1a**)

**B1** (400 mg, 2.0 mmol) was *O*-allylated with allylbromide (968 mg, 0.70 mL, 8.0 mmol) and K_2_CO_3_ (2.21 g, 16 mmol) in acetone (10 mL). The reaction mixture was stirred for 5 h with reflux, and a further 12 h at RT and filtered. The residue was extracted with acetone (50 mL) and the combined organic solutions were evaporated under reduced pressure yielding **B1a** (559 mg, 73%).

Compound **B1a**: IR (ATR, *ν*_max_, cm^−1^): 3025, 2920, 2905, 1595, 1584, 1491, 1450, 1421, 1248, 1116, 1103, 1022, 924, 751 cm^−1^; ^1^H-NMR (CDCl_3_) δ 7.19 (td, *J* = 7.6, 1.5 Hz, 2H, H-4), 7.14 (dd, *J* = 7.5, 1.2 Hz, 2H, H-6), 6.90 (t, *J* ≈ 8 Hz, 2H, H-5), 6.89 (d, 2H, *J* = 7.5 Hz, H-3), 6.02–6.13 (m, 2H, CH_2_–C*H*=CH_2_), 5.42 (dquint, *J* = 17.2, 4.3 Hz, 2H, CH_2_–CH=C*H*_2_), 5.27 (dquint, *J* = 10.5, 1.4 Hz, 2H, CH_2_–CH=C*H*_2_), 4.58 (dq, *J* = 5.0, 1.5 Hz, 2H, C*H*_2_–CH=CH_2_), 4.10 (s, 2H, Ar–C*H*_2_–Ar); ^13^C-NMR (CDCl_3_) δ 156.5 (C-2), 133.6 (*C*H=CH_2_), 130.5 (C-6), 129.6 (C-1), 126.9 (C-4), 120.5 (C-5), 116.8 (CH=*C*H_2_), 111.6 (C-3), 30.8 (O–CH_2_), 29.8 (Ar–CH_2_–Ar); EI^+^ calcd for C_19_H_20_O_2_: [M]^+^ 280.1463; found EI-MS *m*/*z* (rel. int.): 280.1465 [M]^+^ (100). Compound **B1a** has been mentioned in several patents; however, chemical data were not given therein.

2-Allyl-6-(3-allyl-2-hydroxybenzyl)-phenol (**B2**) and 4-Allyl-2-(3-allyl-2-hydroxybenzyl)-phenol (**B3**)

Claisen rearrangement of **B1a** (561 mg, 2 mmol) in *N*,*N*-diethylaniline (4 mL) following the procedure of Chattopadhyay et al. [21] resulted in **B2** (410 mg; 73%) and **B3** (89 mg; 15%) as white wax like solids.

Compound **B2**: IR (KBr, *ν*_max_, cm^−1^): 3407 (br, OH), 3073, 3035, 2975, 2937, 1639, 1590, 1461, 1448, 1372, 1294, 1234, 1210, 1192, 1086, 999, 922, 912, 754 cm^−1^; ^1^H-NMR (CDCl_3_) δ 7.20 (dd, *J* = 7.7, 1.5 Hz, 2H, H-6), 7.02 (d, *J* = 7.7 Hz,2H, H-4), 6.88 (t, *J* = 7.7 Hz, 2H, H-5), 6.43 (s, br, 2H, OH), 6.06 (ddt, *J* = 16.8, 10.3, 6.2 Hz, 2H, CH_2_–C*H*=CH_2_), 5.18–5.24 (m, 4H, CH_2_–CH=C*H*_2_), 3.97 (s, 2H, Ar–C*H*_2_–Ar), 3.45 (d, *J* = 6.6 Hz, 4H, C*H*_2_–CH=CH_2_); ^13^C-NMR (CDCl_3_) δ 151.4 (C-2), 136.6 (CH_2_–C*H*=CH_2_), 129.1 (C-6), 128.7 (C-4), 127.1 (C-1), 125.8 (C-3), 121.1 (C-5), 116.6 (CH_2_–CH=*C*H_2_), 35.4 (*C*H_2_–CH=CH_2_), 30.8 (Ar–CH_2_–Ar); EI^+^ calcd for C_19_H_20_O_2_: [M]^+^ 280.1463; found EI-MS *m*/*z* (rel. int.): 280.1469 [M]^+^ (58). Compound **B2** has been mentioned in several patents; however, chemical data were not given therein.

Compound **B3**: IR (KBr, *ν*_max_, cm^−1^): 3387 (OH), 3077, 2853, 1638, 1611, 1592, 1506, 1465, 1434, 1256, 1232, 1215, 1107, 996, 914, 759 cm^−1^. ^1^H-NMR (CDCl_3_) δ 7.17 (dd, *J* = 7.8, 1.5 Hz, 1H, H-6), 7.06 (d, *J* = 2.2 Hz,1H, H-6′), 6.98 (dd, *J* = 7.3, 1.5 Hz, 1H, H-4), 6.91 (dd, *J* = 8.4, 2.2 Hz, 1H, H-4′), 6.84 (t, *J* = 7.5 Hz, 1H, H-5), 6.74 (d, *J* = 8.0 Hz, 1H, H-3′), 6.31 (s, vb, 2H, OH), 6.02 (ddt, *J* = 16.8, 10.3, 6.2 Hz, 1H, 2″-H), 5.92 (ddt, *J* = 16.9, 10.3, 6.6 Hz, 1H, H-2″), 5.19 (dq, *J* ≈ 16, 1.7 Hz, 1H, H-3″), 5.18 (dq, *J* ≈ 9, 1.5 Hz, 1H, H-3″), 5.03 (dq, *J* ≈ 17, 1.8 Hz, 1H, H-3‴), 5.04 (dq, *J* ≈ 9, 1.3 Hz, 1H, H-3‴), 3.89 (s, 2H, Ar–C*H*_2_–Ar), 3.41 (d, *J* = 6.2 Hz, 2H, H-1″), 3.28 (d, *J* = 7.0 Hz, 2H, H-1‴); ^13^C-NMR (CDCl_3_) δ 151.3 (C-2), 151.2 (C-2′), 137.8 (C-2‴), 136.5 (C-2″), 132.8 (C-5′), 130.8 (C-6′), 129.1 (C-6), 128.8 (C-4), 128.0 (C-4′), 127.1 (C-1), 126.6 (C-1′), 125.6 (C-3), 121.2 (C-5), 116.9 (C-3″), 116.0 (C-3′), 115.4 (C-3‴), 39.4 (C-1‴), 35.6 (C-1″), 30.8 (Ar–*C*H_2_–Ar); EI^+^ calcd for C_19_H_20_O_2_: [M]^+^ 280.1463; found EI-MS *m*/*z* (rel. int.): 280.1464 [M]^+^ (100).

2,4′-Diallyloxy-diphenylmethane (**C1a**) 

**C1** (3.77 g, 18.8 mmol) was allylated with allylbromide (8.97 g, 6.5 mL, 74.1 mmol) and K_2_CO_3_ (20.8 g, 0.15 mol) in acetone (100 mL) as described above yielding the diallyloxy-diphenylmethane (4.12 g, 78%).

Compound **C1a**: IR (ATR, *ν*_max_, cm^−1^): 3077, 3022, 2915, 2860, 1610, 1599, 1584, 1508, 1490, 1451, 1237, 1221, 1020, 996, 921, 749 cm^−1^; ^1^H-NMR (CDCl_3_) δ 7.22 (td, *J* ≈ 8, 1.3 Hz, 1H, H-4), 7.21 (d, *J* ≈ 8 Hz, 2H, H-2′ and H-6′), 7.14 (d, *J* = 7.7 Hz, 1H, H-6), 6.94 (t, *J* = 7.3 Hz, 1H, H-5), 6.90 (d, 1H, *J* ≈ 8 Hz, H-3), 6.89 (d, 2H, *J* = 8.4 Hz, H-3′ and H-5′), 6.03–6.17 (m, 2H, H-2″ and H-2‴), 5.45 (dq, *J* = 17.2, 1.5 Hz, 1H, H-3‴), 5.44 (dq, *J* = 17.3, 1.5 Hz, 1H, H-3″), 5.32 (dq, *J* = 10.5, 1.5 Hz, 1H, H-3‴), 5.31 (dq, *J* = 10.5, 1.5 Hz, 1H, H-3″), 4.58 (dt, *J* = 5.1, 1.5, 2H, H-1″), 4.55 (dt, *J* = 5.1, 1.5, 2H, H-1‴), 4.02 (s, 2H, Ar–C*H*_2_–Ar); ^13^C-NMR (CDCl_3_) δ 156.7(C-4′), 156.2 (C-2), 2 × 133.5 (2 × *C*H=CH_2_), 133.3 (C-1′*), 130.4 (C-1*), 130.2 (C-6), 129.8 (C-2′ and C-6′), 127.2 (C-C-4), 120.6 (C-5), 117.4 (C-3‴), 116.9 (C-3″), 114.5 (C-3′ and C-5′), 111.7 (C-3), 68.8 and 68.7 (2 × O–*C*H_2_), 35.1 (Ar–CH_2_–Ar); EI^+^ calcd for C_19_H_20_O_2_: [M]^+^ 280.1463; found EI-MS *m*/*z* (rel. int.): 280.1 [M]^+^ (45), 133.1 [C_6_H_4_–*O*–allyl]^+^ (100).

2-Allyl-4-(3-allyl-2-hydroxybenzyl)-phenol (**C2**) and 2-Allyl-4-(5-allyl-2-hydroxybenzyl)-phenol (**C3**)

Claisen rearrangement of **C1a** (14.5 g, 51.7 mmol) in *N*,*N*-diethylaniline (115 mL) following the procedure of Chattopadhyay et al. [21] resulted in **C2** (10.6 g; 73%) and **C3** (1.2 g; 8.3%)

Compound **C2**: IR (ATR, *ν*_max_, cm^−1^): 3507 (br, OH), 3076, 3009, 2977, 2906, 1637, 1609, 1591, 1501, 1461, 1432, 1328, 1255, 1182, 996, 911, 751 cm^−1^; ^1^H-NMR (CDCl_3_) δ 6.95–7.06 (m, 4H, H-4,H-6, H-2′, H-6′), 6.86 (t, *J* = 7.5 Hz, 1H, H-5), 6.74 (d, *J* = 7.7 Hz, 1H, H-5′), 5.94–6.09 (m, 2H, H-2″, H-2‴), 5.12–5.20 (m, 4H, H-3″, H-3‴), 4.99 (s, b, 2H, OH), 3.92 (s, 2H, Ar–C*H*_2_–Ar), 3.41 (d, *J* = 6.2 Hz, 2H, H-1″), 3.38 (d, *J* = 6.2 Hz, 2H, H-1‴); ^13^C-NMR (CDCl_3_) δ 152.6 (C-4′), 152.4 (C-2), 136.5 (C-2″), 136.4 (C-2‴), 132.1 (C-1′), 130.7 (C-2′), 129.1 (C-6), 128.6 (C-4), 128.0 (C-6′), 127.6 (C-1), 125.6 (C-3, C-3′), 120.6 (C-5), 116.4 (C-3″, C-3‴), 116.0 (C-5′), 35.7 (Ar–*C*H_2_–Ar), 35.3 (C-1″), 35.1′ (C-1‴); EI^+^ calcd for C_19_H_20_O_2_: [M]^+^ 280.1463; found EI-MS *m*/*z* (rel. int.): 280.1465 [M]^+^ (100).

Compound **C3**: IR (ATR, *ν*_max_, cm^−1^): 3277 (br, OH), 3078, 3013, 2977, 2902, 1638, 1608, 1502, 1434, 1341, 1247, 1207, 1094, 992, 793, 634, 617 cm^−1^; ^1^H-NMR (CDCl_3_) δ 6.90–7.04 (m, 4H, H-4, H-6, H-2′, H-6′), 6.68–6.78 (m 2H, H-3, H-5′), 5.88–6.07 (m, 2H, H-2″, H-2‴), 5.10–5.20 (m, 2H, H-3‴), 4.99–5.10 (m, 2H, H-3″), 4.87 (s, 1H, OH), 4.57 (s, 1H, OH), 3.89 (s, 2H, Ar–C*H*_2_–Ar), 3.38 (d, *J* = 6.2 Hz, 2H, 1‴-H), 3.30 (d, *J* = 6.6 Hz, 2H, H-1′); ^13^C-NMR (CDCl_3_) δ 152.6 (C-4′), 152.1 (C-2), 137.9 (C-2″), 136.4 (C-2‴), 132.2 (C-5), 132.0 (C-1′), 131.0 (C-6), 130.2 (C-2′), 127.9 (C-6′), 127.7 (C-4), 127.1 (C-1), 125.6 (C-3′), 116.3 (C-3‴), 116.0, 115.8 (C-3, C-5′), 115.3 (C-3″), 39.3 (C-1″), 35.7 (Ar–*C*H_2_–Ar), 35.1 (C-1‴ EI^+^ calcd for C_19_H_20_O_2_: [M]^+^ 280.1463; found EI-MS *m*/*z* (rel. int.): 280.1 [M]^+^ (100), 147.1 [CH_2_–C_6_H_4_–*O*–allyl]^+^ (100).

General Procedure for the Methylation of Dihydroxy-Diphenylmethanes

The corresponding diol (1 eq) was dissolved in aqueous KOH (10%, 2.5 eq) and stirred for 10 min. Me_2_SO_4_ (1.2 eq) was added and the resulting mixture was stirred for 20 min at RT and for 1 h at 95 °C. After cooling to RT, the mixture was acidified with aqueous HCl (2M) and extracted with chloroform (3 ×). The organic layers were dried over Na_2_SO_4_ and concentrated under reduced pressure. The residue was chromatographed over silica with cyclohexane/AcOEt = 5:1.

Bis(3-allyl-2-methoxyphenyl)methane (**B4a**) and 6-allyl-2-(3-allyl-2-methoxybenzyl)-phenol (**B4b**)

Methylation of 420 mg (1.5 mmol) **B2** resulted in 39 mg of starting material, 40 mg **B4a** (8.6%) and 249 mg **B4b** (57%).

Compound **B4a**: IR (ATR, *ν*_max_, cm^−1^): 3001, 2978, 2941, 2827, 1463, 1427, 1291, 1164, 1080, 1009, 911, 766 cm^−1^; ^1^H-NMR (CDCl_3_) δ 7.09 (dd, 2H, *J* = 7.6 and 1.3 Hz, H-4), 7.00 (t, 2H, *J* = 7.5 Hz, H-5), 6.93 (dd, 2H, *J* = 7.4 and 1.3 Hz, H-6), 5.97–6.09 (m, 2H, -C*H*=CH_2_), 5.06–5.21 (m, 4H, -CH=C*H*_2_), 4.10 (s, 2H, Ar–C*H*_2_–Ar), 3.72 (s, 6H, OC*H*_3_), 3.46 (d, 4H, *J* 6.6 Hz, C*H*_2_-CH=CH_2_); ^13^C-NMR (CDCl_3_) δ 154.4 (C-2), 137.3 (-*C*H=CH_2_), 134.0 (C-1), 132.9 (C-3), 128.9 (C-6), 128.5 (C-4), 124.1 (C-5), 115.8 (-CH=*C*H_2_), 34.0 (-*C*H_2_-CH=CH_2_), 29.6 (Ar–*C*H_2_–Ar). EI^+^ calcd for C_21_H_24_O_2_: [M]^+^ 308.1776; found EI-MS *m*/*z* (rel. int.): 308.1776 [M]^+^ (100).

Compound **B4b**: IR (ATR, *ν*_max_, cm^−1^): 3320 (br, OH), 3001, 2978, 2975, 2944, 2908, 1641, 1632, 1591, 1462, 1446, 1430, 1220, 1086, 998, 910, 780, 604 cm^−1^; ^1^H-NMR (CDCl_3_) δ 7.35 (s, 1H, OH), 7.18 (dd, *J* = 7.1, 1.8 Hz, 1H, H-6′), 7.13 (d, *J* = 7.1 Hz, 1H, H-6), 7.08 (dd, *J* ≈ 7, ≈ 2 Hz, 1H, H-4′), 7.06 (t, *J* = 7.5 Hz, 1H, H-5′), 7.00 (d, *J* = 7.7 Hz, 1H, H-4), 6.81 (t, *J* = 7.5 Hz, 1H, H-5), 5.92–6.08 (m, 2H, H-2′ and H-2‴), 5.03–5.15 (m, 4H, H-3″ and H-3‴), 3.92 (2 × s, 5H, Ar–C*H*_2_–Ar and OC*H*_3_), 3.47 (d, *J* = 6.3 Hz, 2H, H-1‴), 3.40 (d, *J* = 6.6 Hz, 2H, H-1″); ^13^C-NMR (CDCl_3_) δ 154.2 (C-2′), 151.2 (C-2), 137.0 (C-2″), 136.8 (C-2‴), 2 × 133.0, (C-1′ and C-3′), 129.1 (C-4′), 128.9 (C-6′), 128.6 (C-4), 128.4 (C-6), 127.2 (C-3), 126.6 (C-1), 125.3 (C-5′), 120.1 (C-5), 116.2 (C-3‴), 115.4 (C-3″), 62.3 (O*C*H_3_), 34.7 (C-1″), 33.8 (C-1‴), 31.4 (Ar–*C*H_2_–Ar); EI^+^ calcd for C_21_H_24_O_2_: [M]^+^ 294.1620; found EI-MS *m*/*z* (rel. int.): 294.1619 [M]^+^ (100).

3,3′-Diallyl-2,4′-dimethoxy-diphenyl-methan (**C4a**), 6-allyl-2-(3-allyl-4-methoxybenzyl)-phenol (**C4b**) and 2-allyl-4-(3-allyl-2-methoxybenzyl)-phenol (**C4c**)

Methylation of 2.10 g (7.5 mmol) **C2** resulted in 143 mg of starting material, 318 mg **C4a** (14%), 665 mg **C4b** (30%) and 442 mg **C4c** (20%).

Compound **C4a**: IR (ATR, *ν*_max_, cm^−1^): 3001, 2977, 2939, 2833, 1638, 1500, 1463, 1429, 1248, 1125, 1080, 1033, 1010, 910, 768 cm^−1^; ^1^H-NMR (CDCl_3_) δ 7.07 (dd, *J* = 7.3, 1.5 Hz, 1H, H-4), 6.98–7.02 (m, 3H, H-2′, H-5 and H-6′), 6.96 (dd, *J* = 7.6, 1.3 Hz, 1H, H-6), 6.78 (d, *J* = 8.4 Hz, 1H, H-5′), 5.92–6.07 (m, 2H, H-2″ and H-2‴), 5.06–5.13 (m, 2H, H-3″), 4.99–5.06 (m, 2H, H-3‴), 3.96 (s, 2H, Ar–C*H*_2_–Ar), 3.80 (s, 3H, C4′-OC*H*_3_), 3.68 (s, 3H, C2-OC*H*_3_), 3.46 (d, *J* = 6.3 Hz, 2H, H-1″), 3.36 (d, *J* = 6.6 Hz, 2H, H-1‴); ^13^C-NMR (CDCl_3_) δ 156.3 (C-2), 155.6 (C-2′), 137.3 (C-2″), 137.1 (C-2‴), 134.7 (C-1), 133.0 (C-3), 132.8 (C-1′), 130.2 (C-2′), 129.0 (C-6), 128.5 (C-4), 128.4 (C-3′), 127.5 (C-6′), 120.5 (C-5), 115.8 (C-3″), 115.3 (C-3‴), 110.3 (C-5′), 61.2 (C2-O*C*H_3_), 55.4 (C4′-O*C*H_3_), 34.9 (Ar–*C*H_2_–Ar), 34.3 (C-1‴), 34.0 (C-1″); MS (ESI^+^) *m*/*z* (%): calculated for C_21_H_24_O_2_: [M]^+^ 308.178; found: EI-MS *m*/*z* (rel. int.): 308.4 [M]^+^ (100).

**D4b**: IR (ATR, *ν*_max_, cm^−1^): 3526 (br, OH), 3003, 2976, 2905, 2834, 1637, 1608, 1592, 1499, 1461, 1247, 1182, 1032, 996, 910, 752 cm^−1^; ^1^H-NMR (CDCl_3_) δ 6.97–7.02 (m, 4H, H-4, H-6, H-2′ and H-6′), 6.82 (t, *J* = 7.5 Hz, 1H, H-5), 6.76 (d, *J* = 8.8 Hz, 1H, H-5′), 5.90–6.05 (m, 2H, H-2″and H-2‴), 5.11–5.17 (m, 2H, H-3″), 4.98–5.05 (m, 2H, H-3‴), 4.94 (s, 1H, OH), 3.91 (s, 2H, Ar–C*H*_2_–Ar), 3.78 (s, 3H, OC*H*_3_), 3.38 (d, *J* = 6.2 Hz, 2H, H-1″), 3.34 (d, *J* = 6.5 Hz, 2H, H-1‴); ^13^C-NMR (CDCl_3_) δ 152.9 (C-4′), 152.4 (C-2), 136.9 (C-2‴), 136.5 (C-2″), 131.4 (C-1′), 130.2 (C-2′), 129.1 (C-6′), 128.8 (C-3′), 128. 6 (C-4), 127.6 (C-1), 127.2 (C-6), 125.5 (C-3), 120.5 (C-5), 116.4 (C-3″), 115.3 (C-3‴), 55.4 (O*C*H_3_), 35.8 (Ar–*C*H_2_–Ar), 35.3 (C-1″), 34.3 (C-1‴); MS (EI) *m*/*z* (%): calculated for C_20_H_22_O_2_: [M]^+^ 294.162; found: EI-MS *m*/*z* (rel. int.): 294.4 [M]^+^ (80), 253.2 (65).

**D4c**: IR (ATR, *ν*_max_, cm^−1^): 3393 (br, OH), 3005, 2977, 2940, 2911, 2830, 1638, 1610, 1591, 1505, 1464, 1428, 1251, 1198, 1080, 996, 910, 768 cm^−1^; ^1^H-NMR (CDCl_3_) δ 7.09 (dd, *J* = 7.3, 1.7 Hz, 1H, H-4), 7.01 (t, *J* = 7.3 Hz, 1H, H-5), 6.93–6.99 (m, 3H, H-2′, H-6 and H-6′), 6.73 (d, *J* = 7.7 Hz, 1H, H-5′), 5.94–6.08 (m, 2H, H-2″ and H-2‴), 5.06–5.20 (m, 4H, H-3″ and H-3‴), 4.95 (s, 1H, OH), 3.96 (s, 2H, Ar–C*H*_2_–Ar), 3.68 (s, 3H, OC*H*_3_), 3.46 (d, *J* = 6.6 Hz, 2H, H-1″), 3.38 (d, *J* = 6.5 Hz, 2H, H-1‴), ^13^C-NMR (CDCl_3_) δ 156.3 (C-2), 152.3 (C-4′), 137.3 (C-2″), 136.5 (C-2‴), 134.6 (C-1), 133.3 (C-1′), 133.0 (C-3), 130.1 (C-2′), 129.0 (C-6), 128.6 (C-4), 128.2 (C-6′), 125.1 (C-3′), 124.1 (C-5), 116.3 (C-3″), 115.8 (C-3‴), 115.7 (C-5′), 61.2 (O*C*H_3_), 35.2 (C-1‴), 34.9 (Ar–*C*H_2_–Ar), 33.9 (C-1″); MS (EI) *m*/*z* (%): calculated for C_20_H_22_O_2_: [M]^+^ 294.162; found: EI-MS *m*/*z* (rel. int.): 294.4 [M]^+^ (75), 253.2 (40).

### 3.2. Biological Assays

*Cell line:* All screening was done in a line of human embryonic kidney cells stably expressing the human norepinephrine transporter and the vesicular monoamine transporter 2 (HEK-hNET- -hVMAT2-mCherry). Human VMAT2 expression, in addition to hNET, more fully recapitulates a neuronal phenotype in cell culture. Cells were imaged and fully characterized for functionality (Figure 1). For imaging, cells were fixed in 4% paraformaldehyde and protein expression of NET determined by immunolabelling (primary antibody: Novus, anti-norepinephrine transporter, 1:1000; secondary antibody: Thermo Fisher, Alexafluor goat anti-rabbit-488, 1:5000) followed by confocal imaging (Nikon A1R, Nikon Instruments, Melville, NY, USA). *Screening:* Synthesized compounds were tested for interaction with NET via a three-step process. First, all compounds were tested using a commercially available fluorescent assay (neurotransmitter transport uptake kit, Molecular Devices, San Jose, CA, USA) for the assessment of plasmalemmal monoamine transport ability [23,24]. Cells were plated in poly-d-lysine (PDL)-coated 96-well plates (Corning Inc., Corning, NY, USA) to 100% confluence. The following day, cell media (DMEM, 10% fetal bovine serum, 1% penicillin/streptomycin) was removed, and experimental media (Hank’s balanced salt solution, Sigma-Aldrich) was added. Following a 30-min incubation at 37 °C, dye was added to all wells, with or without compounds (3 nM–100 μM final concentration) or selective NET inhibitor desipramine (3 nM–10 μM final concentration, control). Plates were incubated for 1 h, then read on a fluorescent plate reader (Thermo Fisher). Experiments were conducted in triplicate. Second, the most potent modulators of transport activity (**C4a**, **H8**, **H11**, **H16**, **H20** and **M6**) were further assessed for radiolabeled dopamine uptake in the same cell line, as dopamine is a substrate of NET [25,26]. Cells were plated to confluence in 48-well PDL-coated plates the day prior to the experiment. For experiments, media was removed, cells were rinsed with phosphate buffered saline (PBS), and incubated with or without compounds or desipramine (10 nM to 10 μM, non-specific uptake measured by addition of 100 μM desipramine) for ten minutes at 37 °C. Dopamine (1 μM total concentration with 200 nM ^3^H-dopamine tracer) was added and uptake proceeded for 10 min at 37 °C. Cells were then rinsed three times with PBS, lysed with 0.1 N NaOH, and measured via liquid scintillation counting (Beckman Coulter, Brea, CA, USA). Finally, each selected compound was visualized with high-content imaging using the Array Scan VTI HCS (Cellomics). Cells were seeded to 80% confluence in PDL-coated, glass-bottom 96-well plates. Media was removed and DMEM without phenol red with or without compounds or desipramine (10 nM to 10 μM) was added. Following a one-hour incubation, cells were imaged. Forty-nine contiguous fields per well were taken and total fluorescence in the green channel was reported. No compound demonstrated potent activity at NET (IC_50_ > 100 µM), as compared with the selective NET inhibitor, desipramine, which inhibited fluorescent (plate reader: IC_50_ = 125.3 nM; imaging: IC_50_ = 65.7 nM, Figures 1.1 and 3) and radioactive uptake (IC_50_ = 52.1 nM, Figure 1.2). Dose-Response curves (measured as fluorescence) for tested compounds can be found in “Supplementary Materials”.

## 4. Conclusions

Altogether, we tested a series of 52 biphenyl neolignans and derivatives (including diphenyl-methane analogs) found in biological preparations that are used to treat anxiety and insomnia in Eastern medicine, for pharmacological efficacy at the NET. No tested compound potently inhibited uptake via NET, suggesting acutely that these compounds may work exclusively through GABA_A_ receptor modulation. It is also possible that these compounds still modulate the noradrenergic system; long-term treatment with the compounds could change tissue expression of receptors or affect the tissue level of norepinephrine, potentially by modulating enzymes that produce and metabolize the neurotransmitter. Future experiments are needed to determine if chronic administration of drugs may change noradrenergic signaling. Within the context of these experi-ments, however, we see no evidence for acute modulation of NET activity by biphenyl neolignans or derivatives. It is also of note that serotonergic signaling also plays a major role in anxiety and depression. Though beyond the scope of this work, investigation of honokiol derivatives at serotonin receptors may provide further information as to how these compounds exert their anxiolytic effect.

## Figures and Tables

**Table 1:**
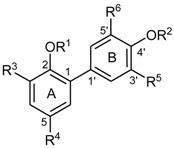
Structures of Compounds Based on Honokiol.

Cpd.	R^1^	R^2^	R^3^	R^4^	R^5^	R^6^
**H**	H	H	H	allyl	allyl	H
**H1**	H	Me	NH_2_	allyl	allyl	H
**H2**	H	Me	NH-COC_11_H_23_	allyl	allyl	H
**H3**	H	Me	NH-Ala	allyl	allyl	H
**H4**	H	Me	NH-Phe	allyl	allyl	H
**H5**	H	Et	NH_2_	allyl	allyl	H
**H6**	Et	Me	NH_2_	allyl	allyl	H
**H7**	Et	Me	NH-Ac	allyl	allyl	H
**H8**	H	Me	NH-Ac	allyl	allyl	H
**H9**	Me	H	H	allyl	allyl	NH-Ac
**H10**	H	Et	H	allyl	allyl	H
**H11**	H	Me	H	allyl	allyl	H
**H12**	Me	H	H	allyl	allyl	NH_2_
**H13**	H	H	H	allyl	1-propenyl	H
**H14**	H	H	H	*n*-propyl	*n*-propyl	H
**H15**	Me	H	H	allyl	allyl	H
**H16**	Me	Me	H	allyl	allyl	H
**H17**	H	Me	H	allyl	1-propenyl	H
**H18**	H	Me	H	2,3-dihydroxypropyl	2,3-dihydroxypropyl	H
**H19**	H	Me	H	*n*-propyl	*n*-propyl	H
**H20**	Me	Me	H	*n*-propyl	*n*-propyl	H
**H21**	Et	H	H	allyl	allyl	H
**H22**	H	Et	H	allyl	allyl	H
**H23**	Et	H	H	*n*-propyl	*n*-propyl	H
**H24**	Et	Et	H	*n*-propyl	*n*-propyl	H
**H25**	H	Me	H	2-bromopropyl	allyl	H
**H26**	Me	H	H	allyl	allyl	NO_2_
**H27**	Me	H	H	*n*-propyl	*n*-propyl	NO_2_
**H28**	H	Me	NO_2_	allyl	allyl	H
**H29**	Me	H	H	*n*-propyl	*n*-propyl	NH_2_
**H30**	H	Me	NH_2_	*n*-propyl	*n*-propyl	H
**H31**	Me	H	H	*n*-propyl	*n*-propyl	NH-Ac
**H32**	H	Me	NH-Ac	*n*-propyl	*n*-propyl	H

**Table 2:**
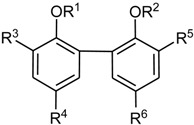
Structures of Compounds Based on Biphenyl-2,2′-diol.

Cpd.	R^1^	R^2^	R^3^	R^4^	R^5^	R^6^
**M**	H	H	H	allyl	H	allyl
**M1**	H	H	H	H	H	H
**M2**	H	H	H	*n*-propyl	H	*n*-propyl
**M3**	Me	H	H	allyl	H	allyl
**M4**	Me	Me	H	allyl	H	allyl
**M5**	H	H	H	Me	H	Me
**M6**	H	H	H	*t*-butyl	H	*t*-butyl
**M7**	H	H	allyl	H	allyl	H

**Table 3:**
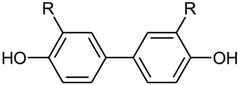
Structures of Compounds Based on Biphenyl-4,4′-diol.

Cpd.	R
**A1**	H
**A2**	allyl

**Table 4:**
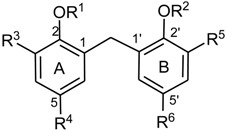
Structures of Compounds Based on Bis(2-hydroxyphenyl)methane.

Cpd.	R^1^	R^2^	R^3^	R^4^	R^5^	R^6^
**B1**	H	H	H	H	H	H
B1a *	allyl	allyl	H	H	H	H
**B2**	H	H	allyl	H	allyl	H
B3 *	H	H	allyl	H	H	allyl
**B4b**	Me	H	allyl	H	allyl	H
**B4a**	Me	Me	allyl	H	allyl	H

* Compounds not tested.

**Table 5:**
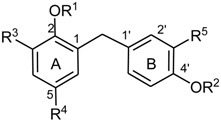
Structures of Compounds Based on 2,4′-Dihydroxydiphenylmethane.

Cpd.	R^1^	R^2^	R^3^	R^4^	R^5^
**C1**	H	H	H	H	H
C1a *	allyl	allyl	H	H	H
**C2**	H	H	allyl	H	allyl
C3 *	H	H	H	allyl	allyl
**C4c**	Me	H	allyl	H	allyl
**C4b**	H	Me	allyl	H	allyl
**C4a**	Me	Me	allyl	H	allyl

* Compounds not tested.

## Supplementary Materials

Proton NMR spectra of the new compounds and dose-response curves of the tested compounds are available in “Supplementary Materials”.
