# Supplementary material for: Bioinspired Honokiol Analogs and Their Evaluation for Activity on the Norepinephrine Transporter
Source: Molecules. 2018 Oct 4;23(10):2536. doi: 10.3390/molecules23102536 (PMC6222891; doi:10.3390/molecules23102536)
Supplement: Supplementary file 1 [file molecules-23-02536-s001.pdf]

SUPPLEMENTAL MATERIAL TO:

Bioinspired Honokiol Analogs and Their Evaluation for Activity on the Norepinephrine Transporter

Kristen Stout[1,2], Marketa Bernaskova[3], Gary W. Miller[1,2], Antje Huefner[3] and Wolfgang Schuehly[4,5]

[1] Rollins School of Public Health, Emory University, 1518 Clifton Road, NE, Claudia Nance Rollins Bldg, Atlanta, GA 30322 (USA)

[2] Columbia University Mailman School of Public Health, 722 West 168th Street, Room 1411B, New York, NY 10032 (USA)

[3] Institute of Pharmaceutical Sciences, Pharmaceutical Chemistry, Universitätsplatz 1, University of Graz, 8010 Graz, Austria

[4] Institute of Pharmaceutical Sciences, Pharmacognosy, Universitätsplatz 4, University of Graz, 8010 Graz, Austria

[5] Institute of Biology, Universitätsplatz 2, University of Graz, 8010 Graz, Austria

Content of supporting information file:

1. ^{1}H-NMR spectra ($CDCl_3$, 400 MHz) of the new compounds (page 3-12)

2. Dose-Response (measured as fluorescence) curves for tested compounds (page 13-23)

1. ^{1}H-NMR spectra of the new compounds

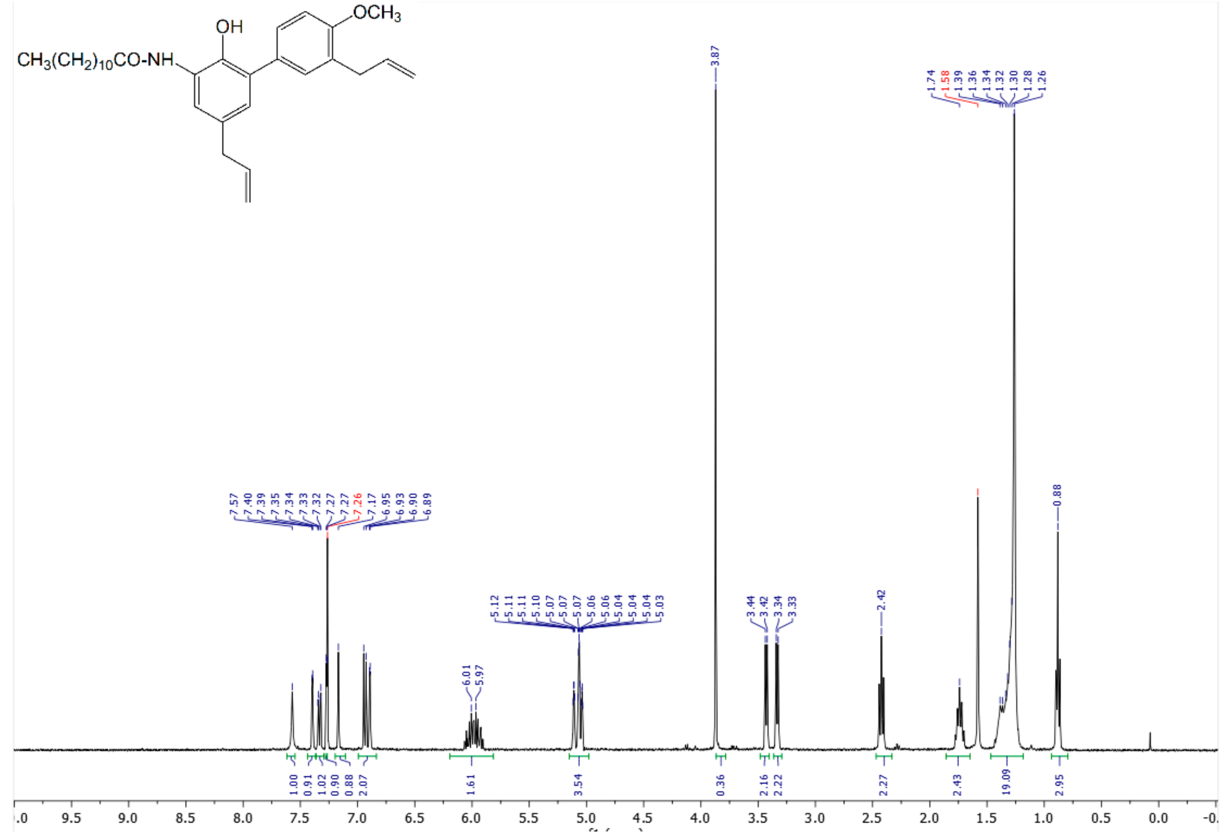

^{1}H-NMR spectrum ($CDCl_3$, 400 MHz) of **H2**

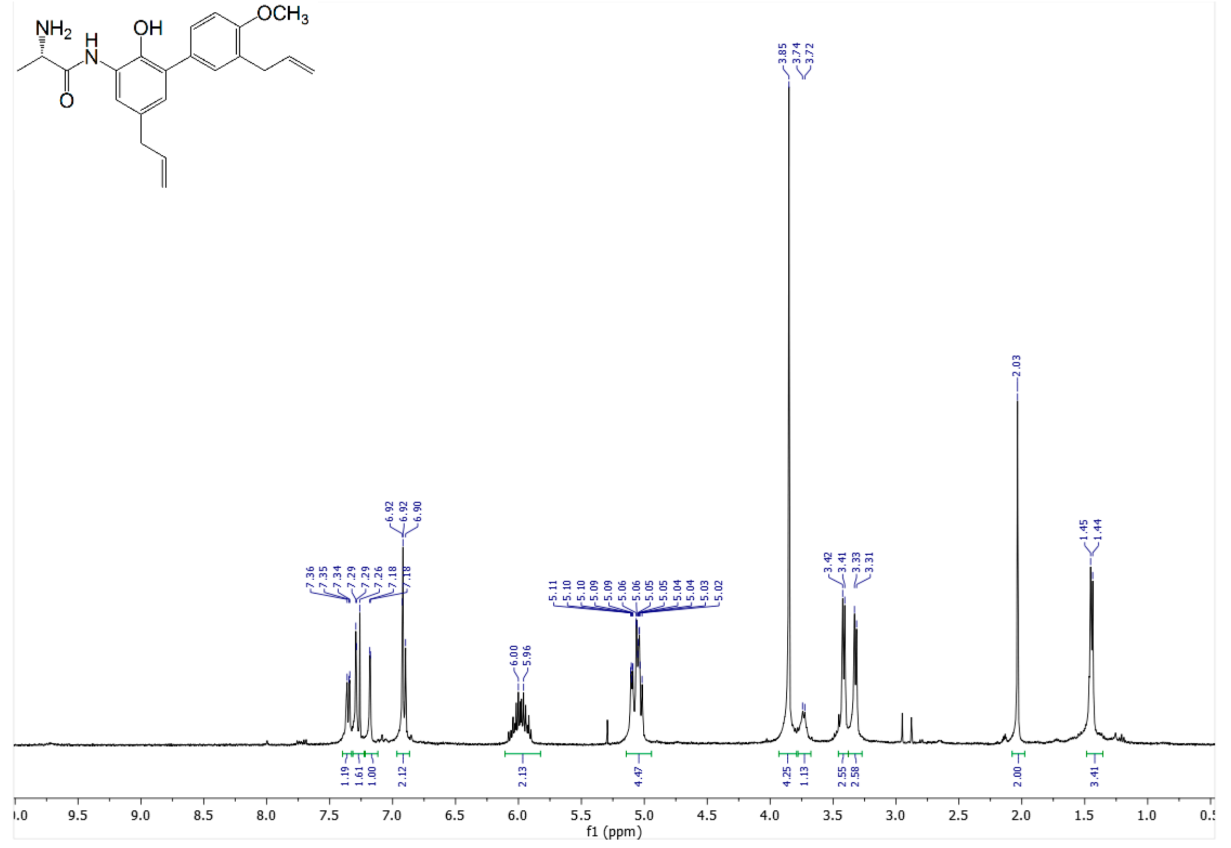

H-NMR spectrum ($CDCl_3/H_2O$, 400 MHz) of **H3**

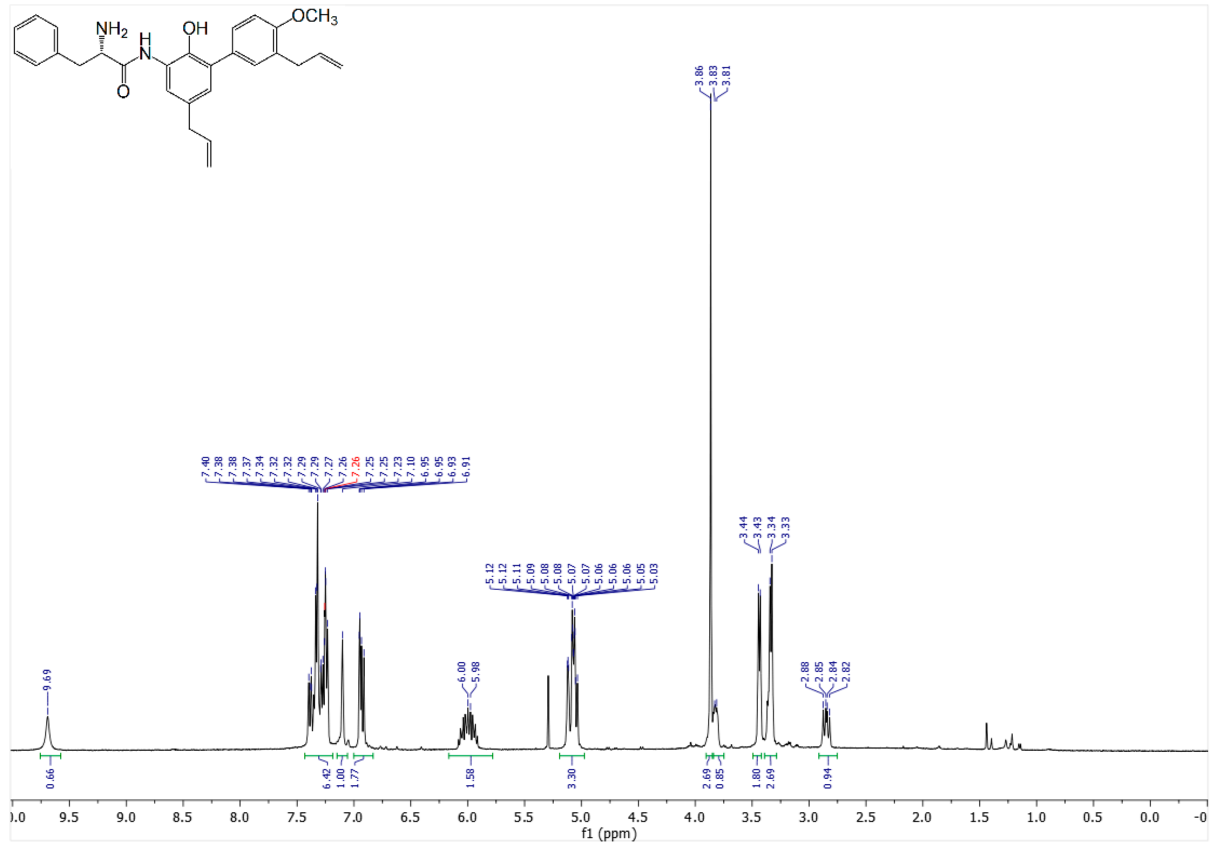

^{1}H-NMR spectrum ($CDCl_3/H_2O$, 400 MHz) of **H4**

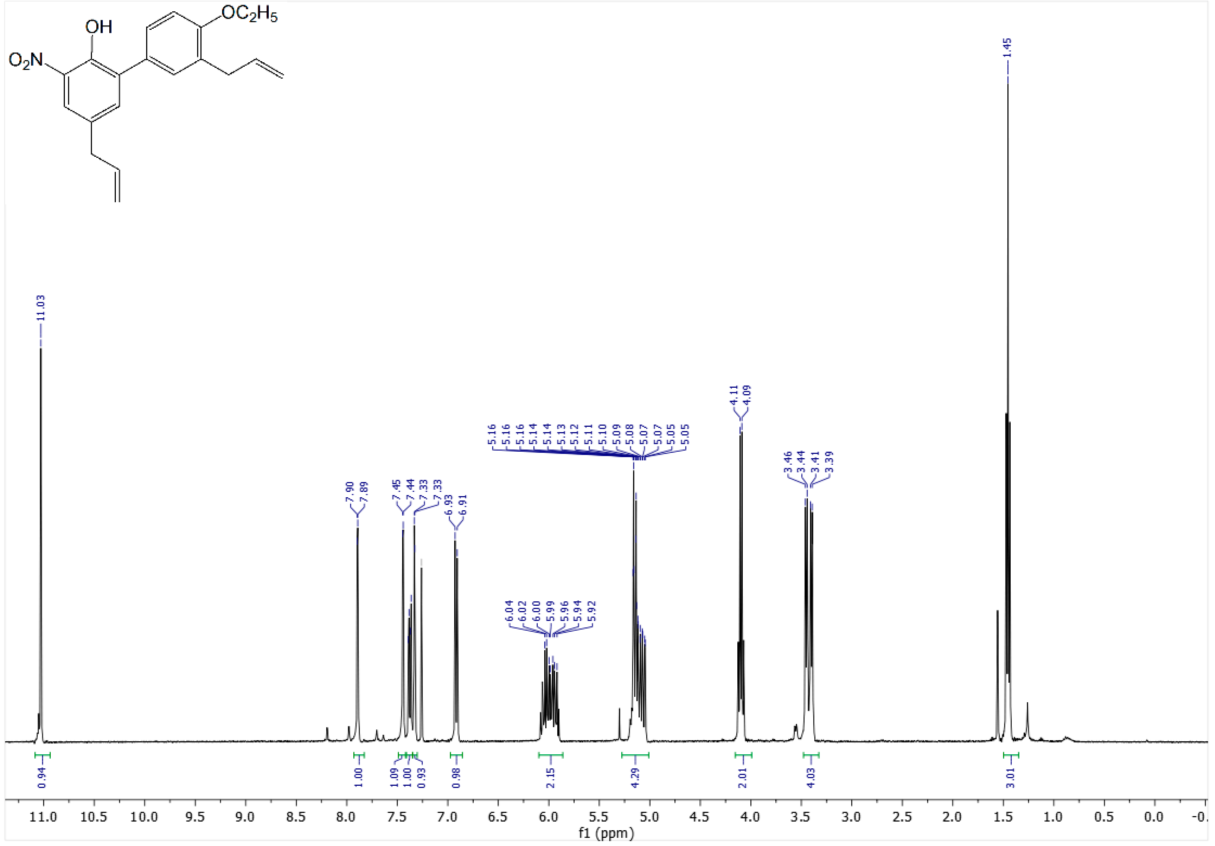

^{1}H-NMR spectrum ($CDCl_3$, 400 MHz) of **H5a**

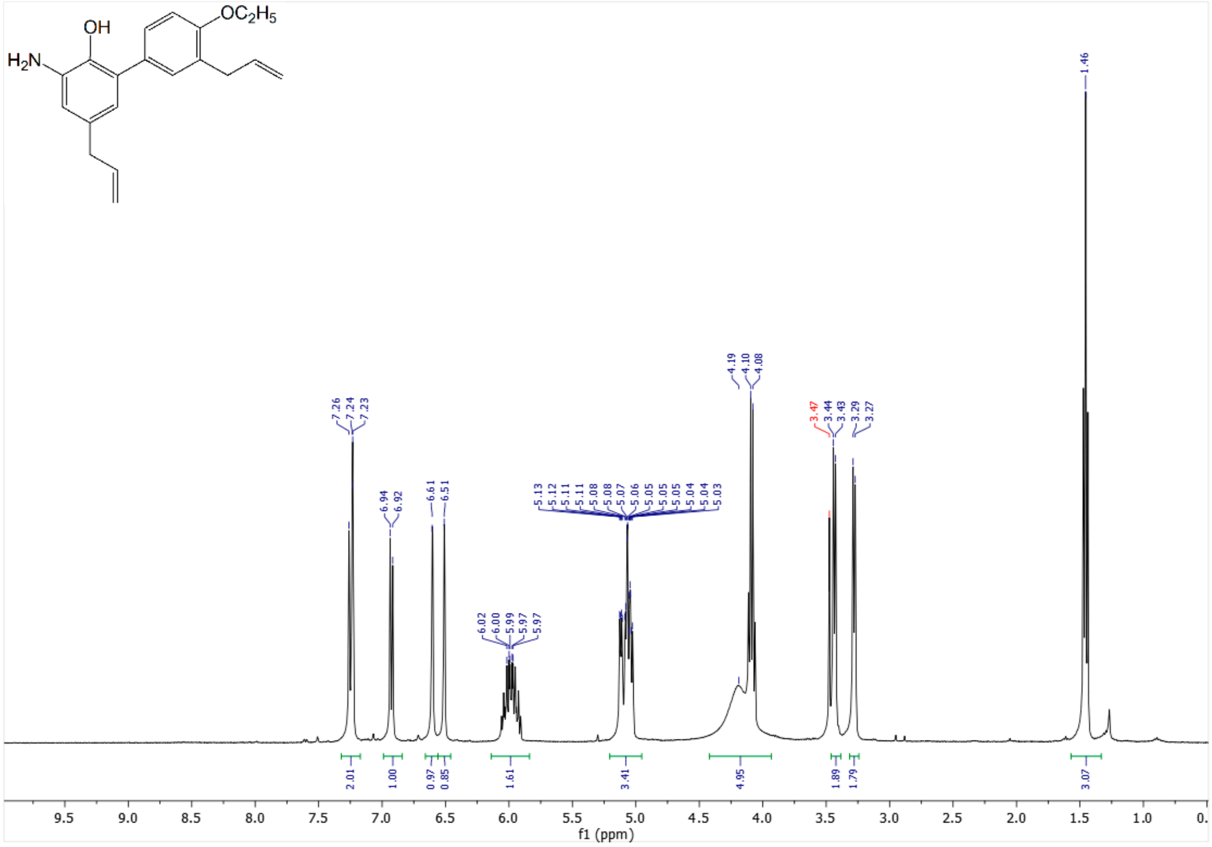

^{1}H-NMR spectrum (CDCl3, 400 MHz) of **H5**

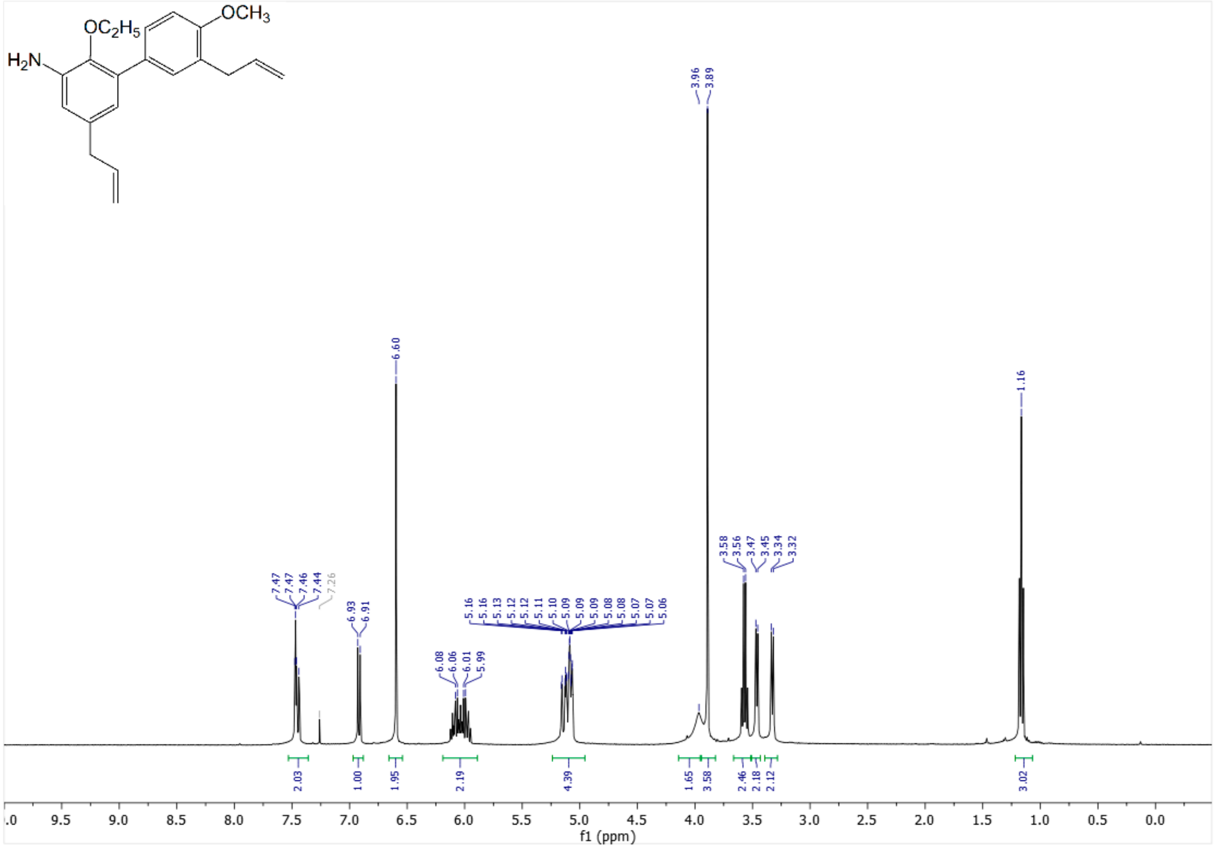

^{1}H-NMR spectrum (CDCl3, 400 MHz) of **H6**

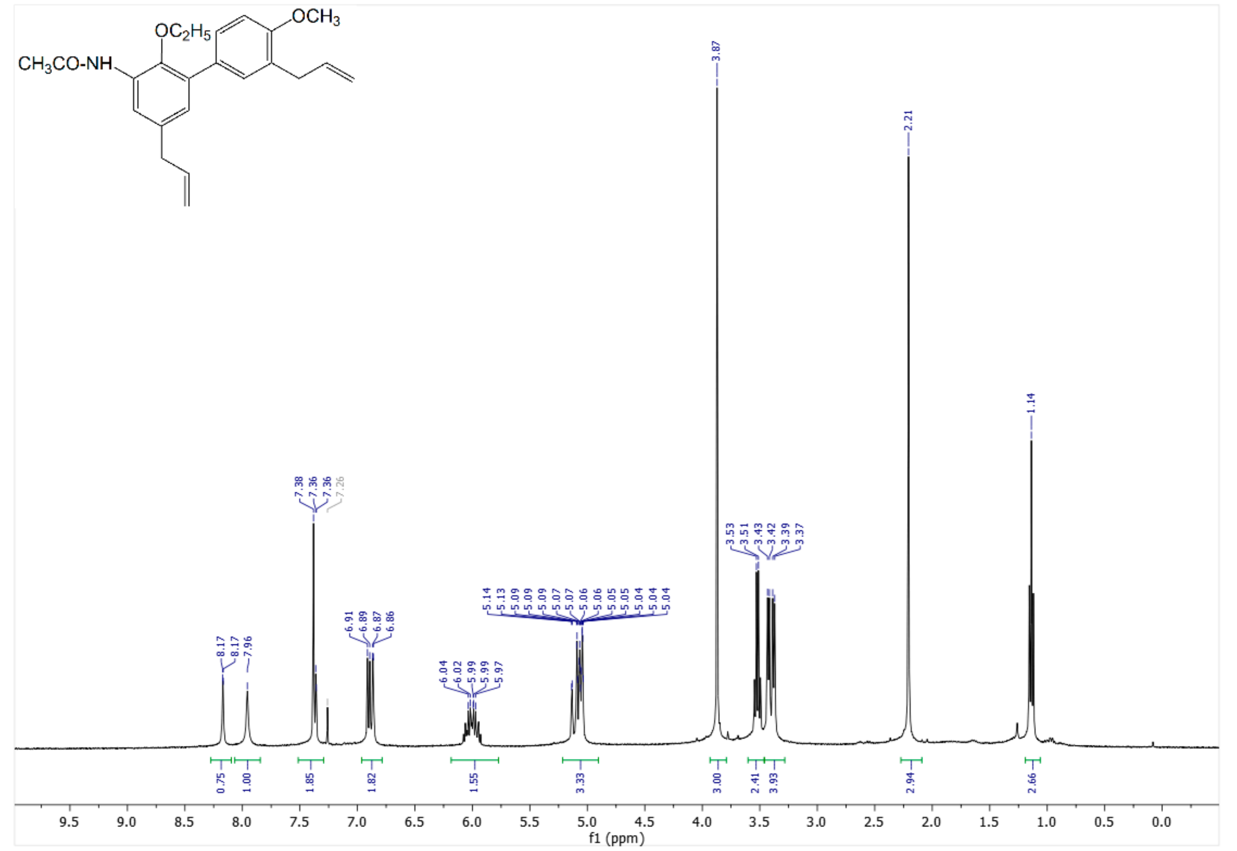

^{1}H-NMR spectrum ($CDCl_3$, 400 MHz) of **H7**

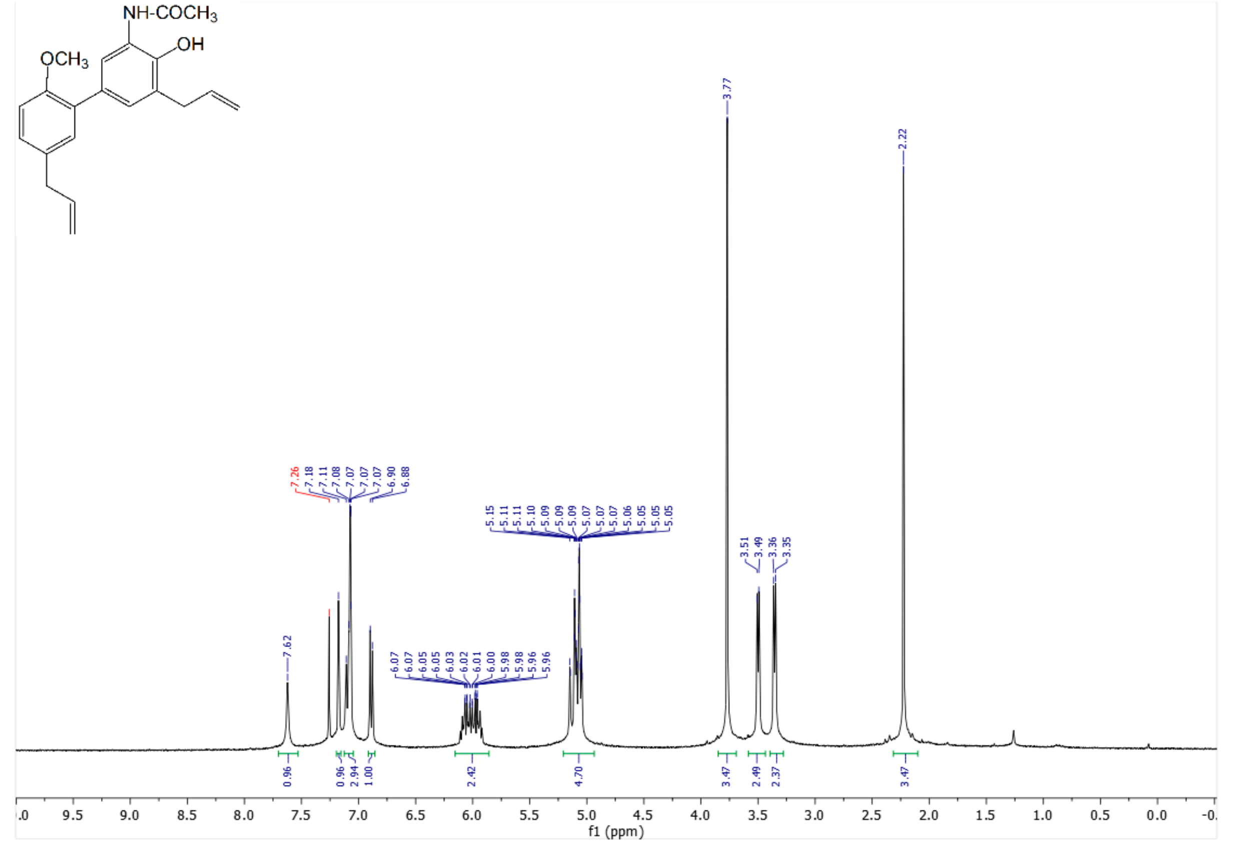

^{1}H-NMR spectrum ($CDCl_3$, 400 MHz) of **H9**

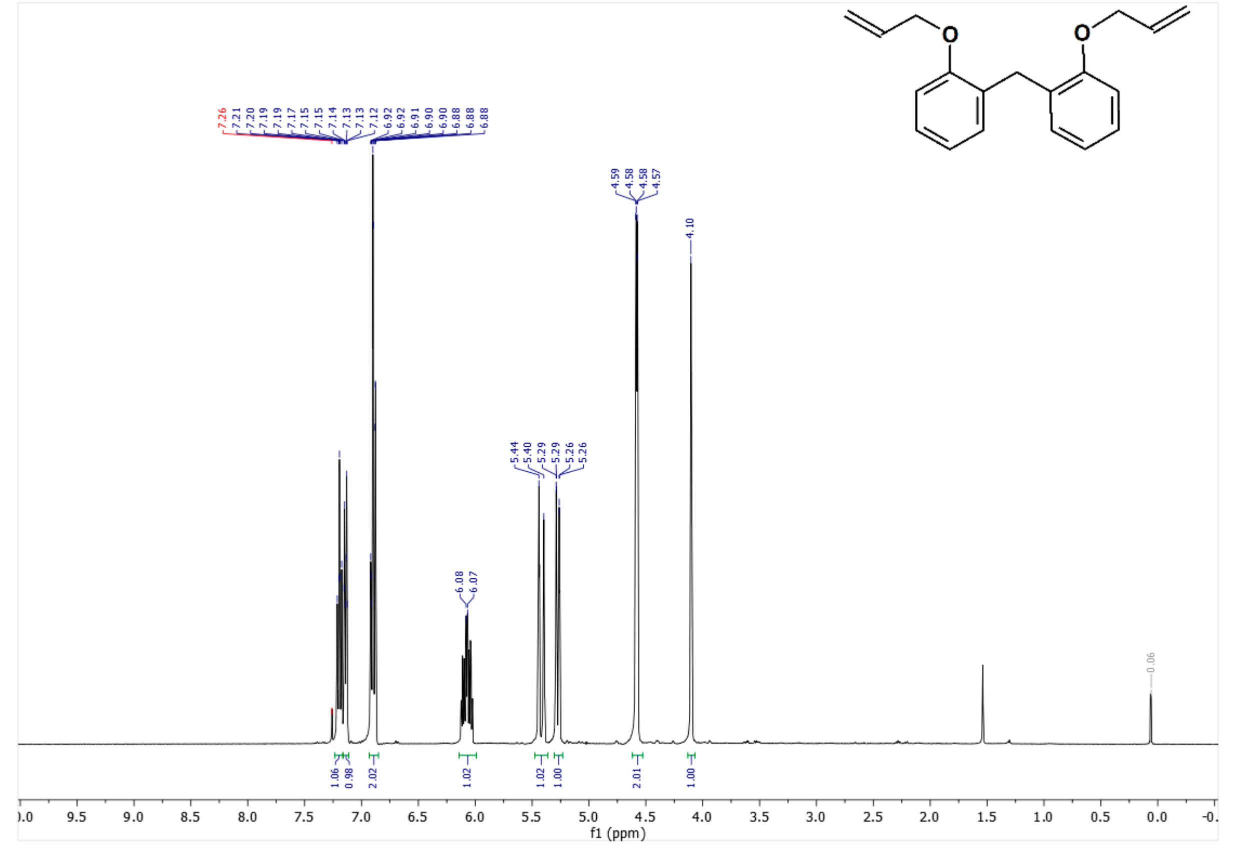

^{1}H-NMR spectrum (CDCl$_3$, 400 MHz) of **B1a**

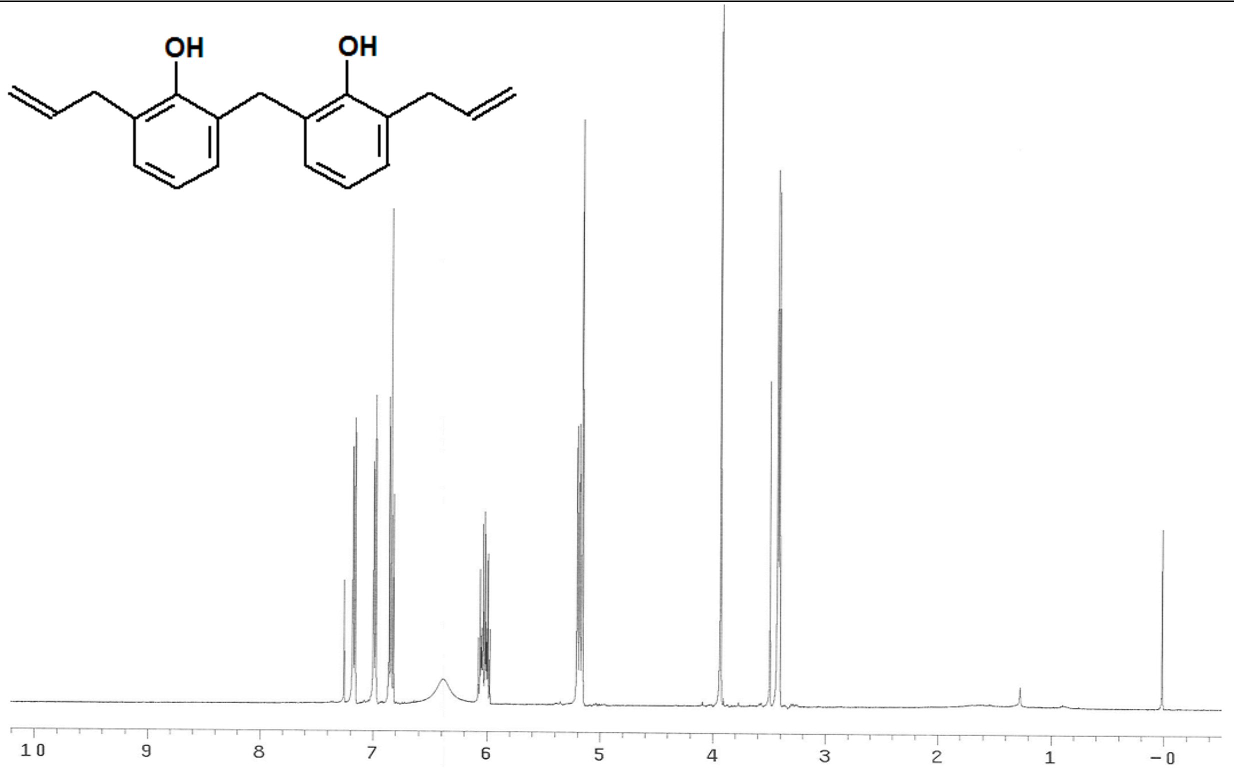

^{1}H-NMR spectrum (CDCl$_3$, 400 MHz) of **B2**

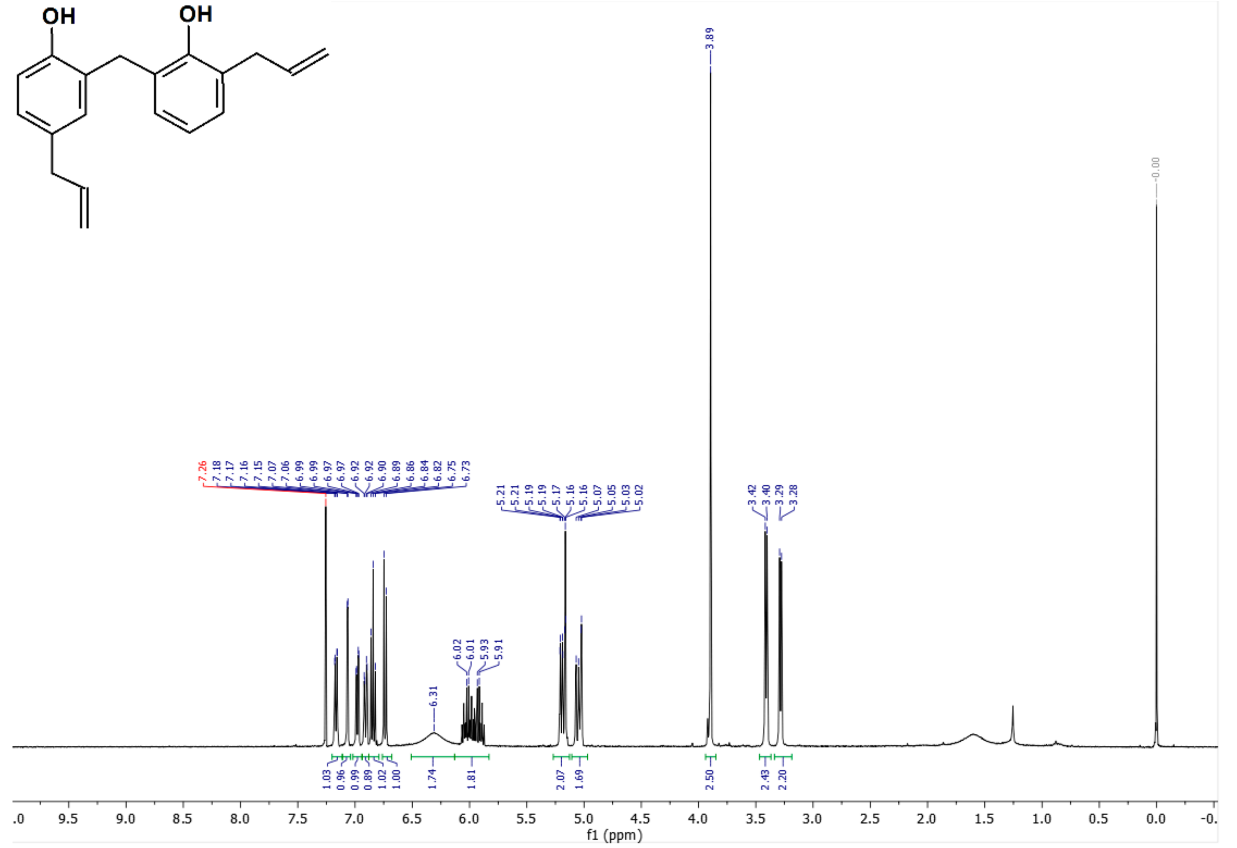

^{1}H-NMR spectrum ($CDCl_3$, 400 MHz) of **B3**

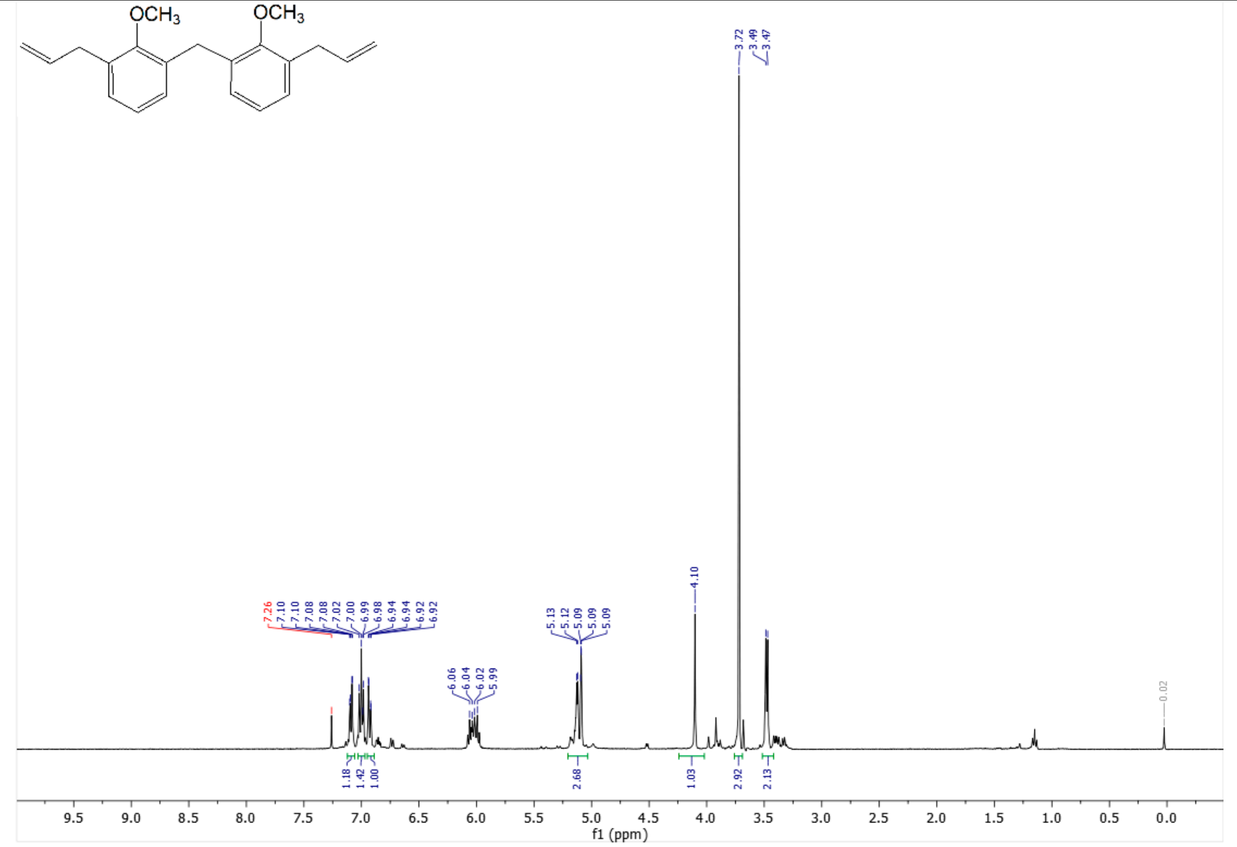

^{1}H-NMR spectrum ($CDCl_3$, 400 MHz) of **B4a**

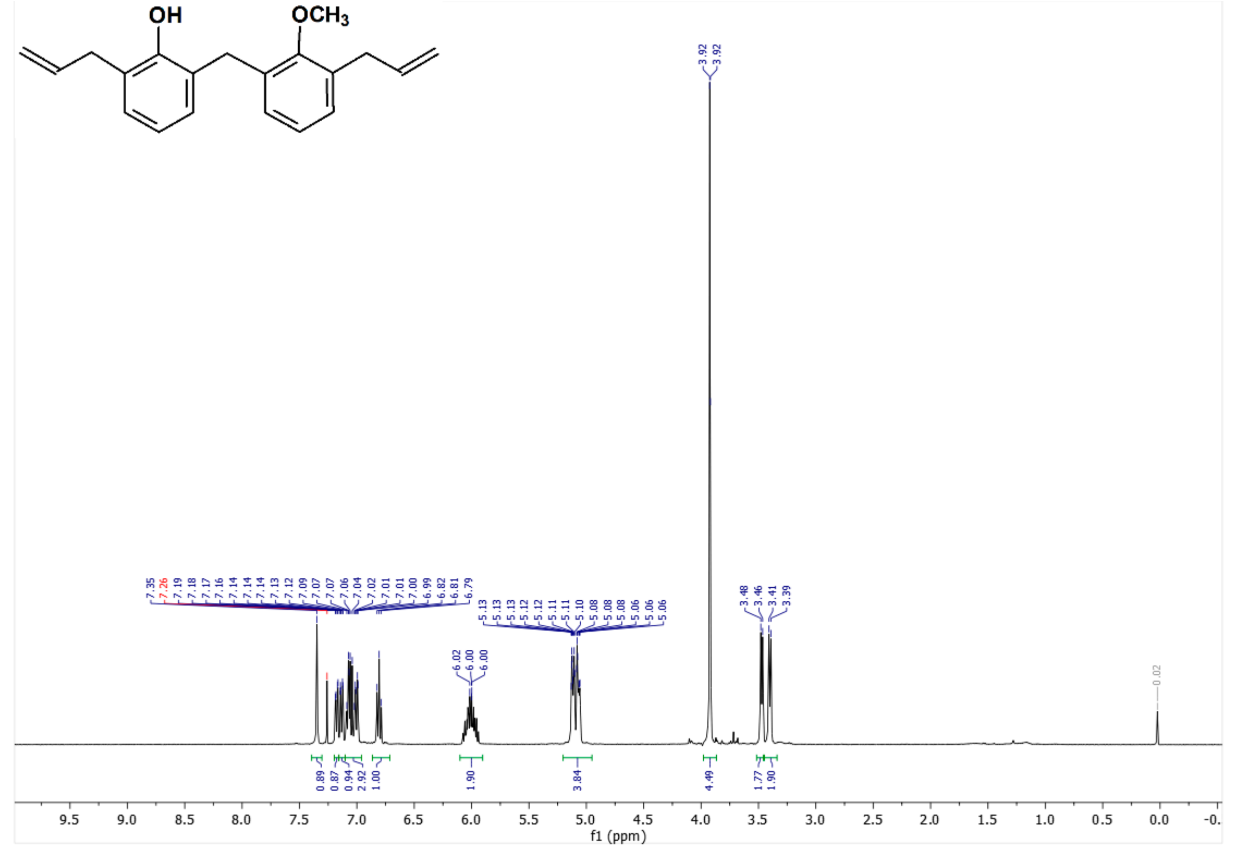

^{1}H-NMR spectrum ($CDCl_3$, 400 MHz) of **B4b**

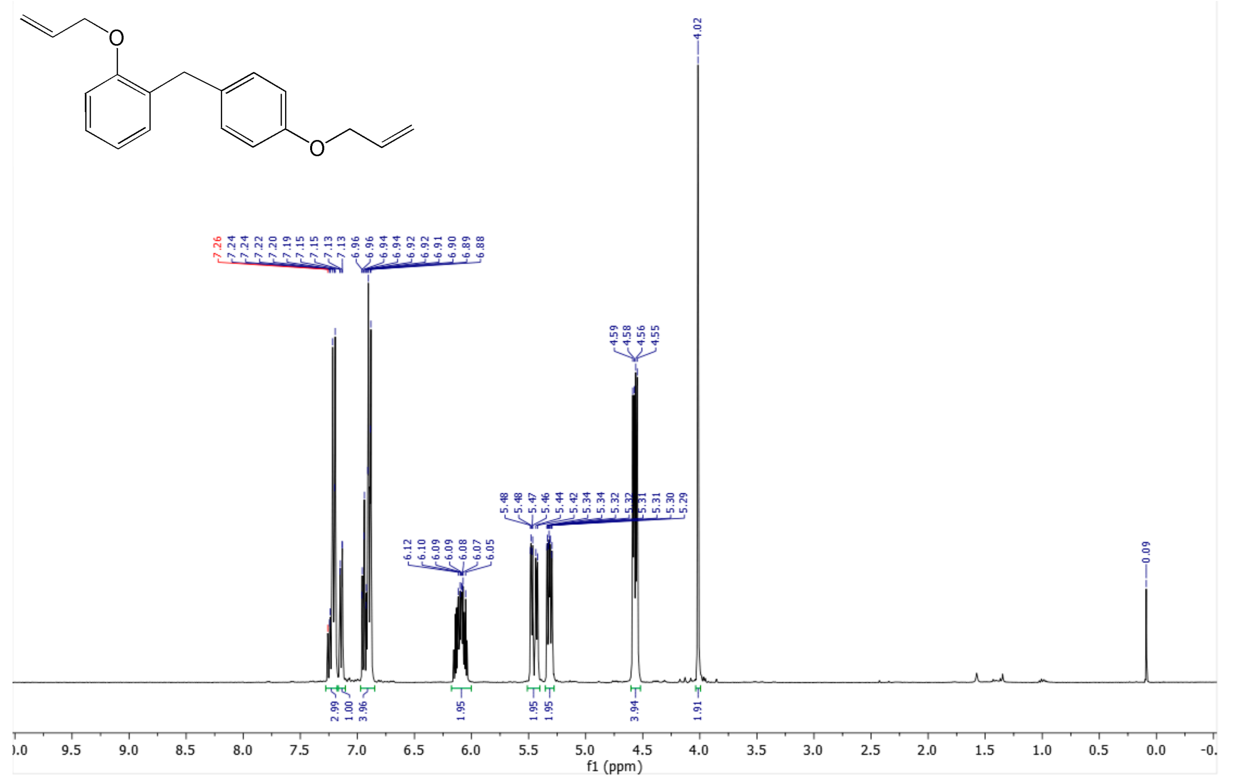

^{1}H-NMR spectrum ($CDCl_3$, 400 MHz) of **C1a**

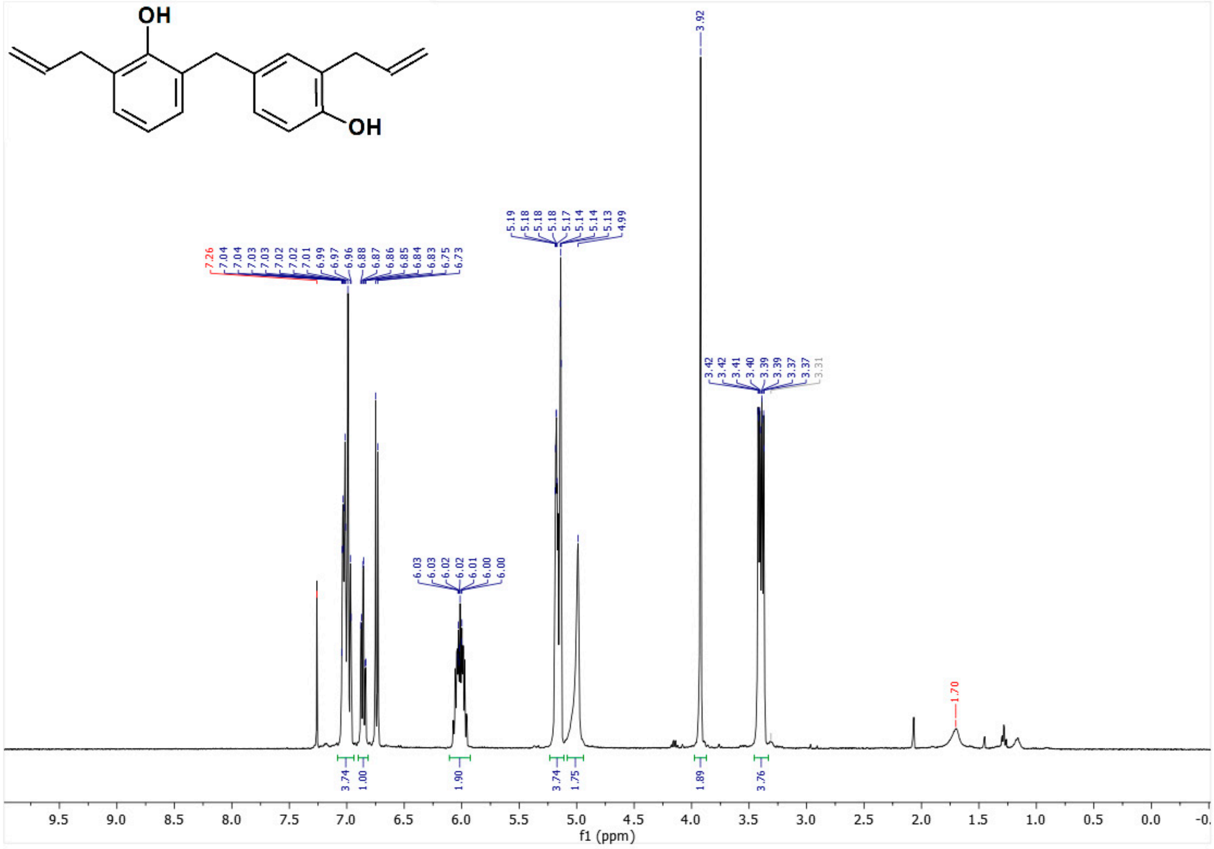

^{1}H-NMR spectrum of (CDCl$_3$, 400 MHz) **C2**

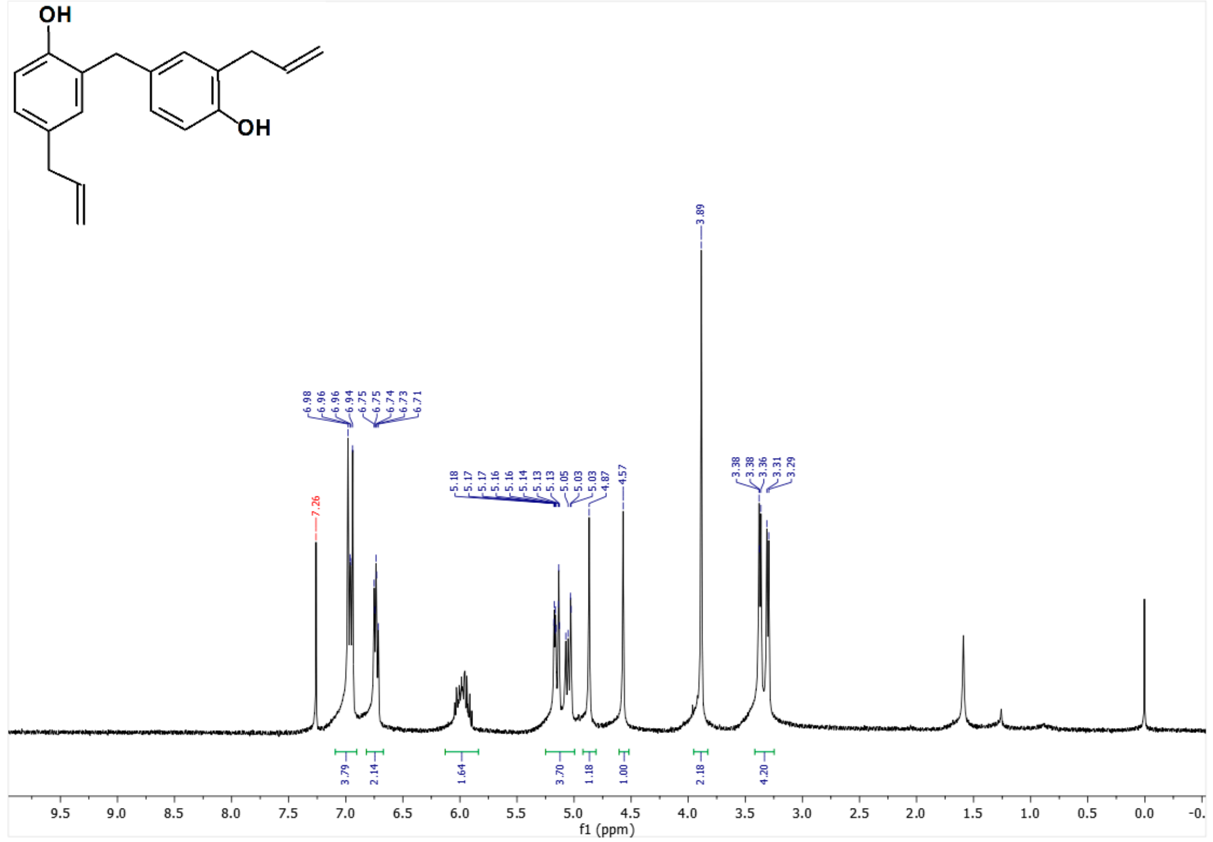

^{1}H-NMR spectrum (CDCl$_3$, 400 MHz) of **C3**

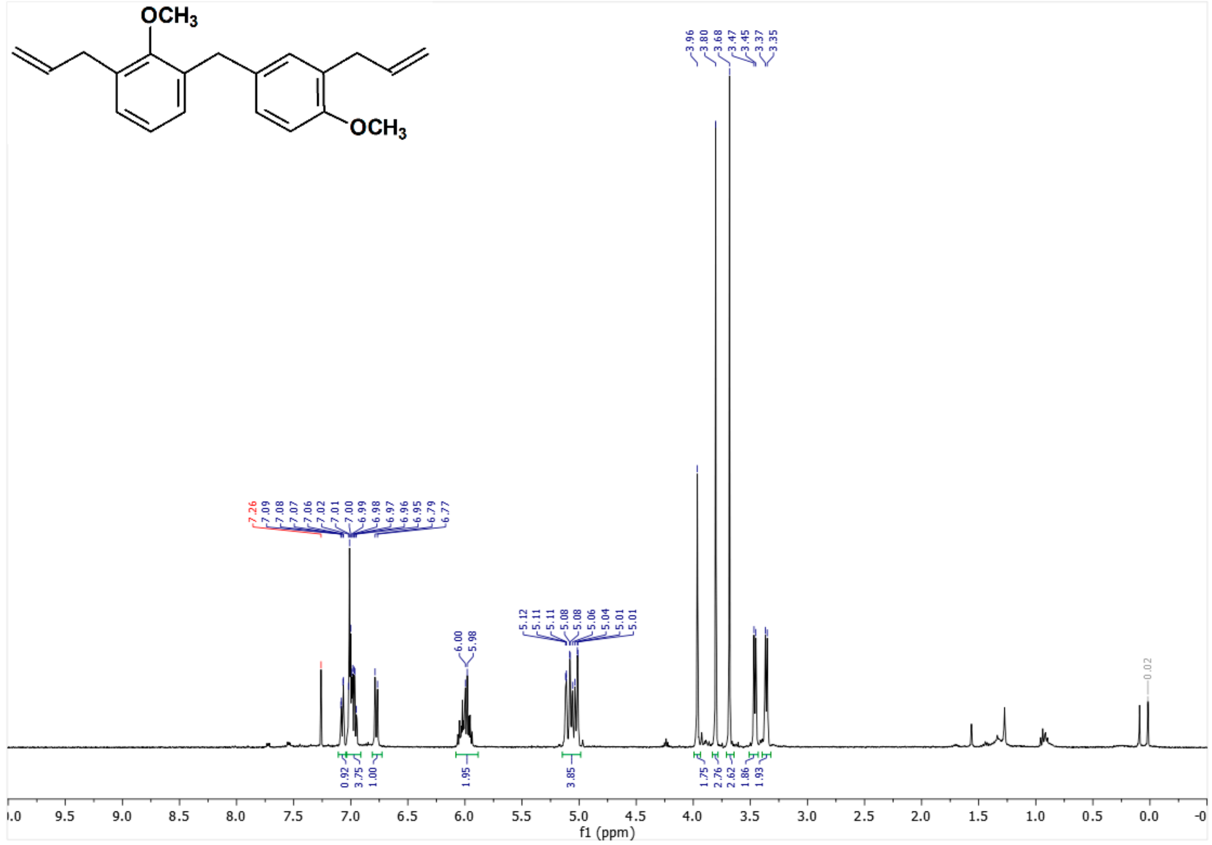

^{1}H-NMR spectrum ($CDCl_3$, 400 MHz) of **C4a**

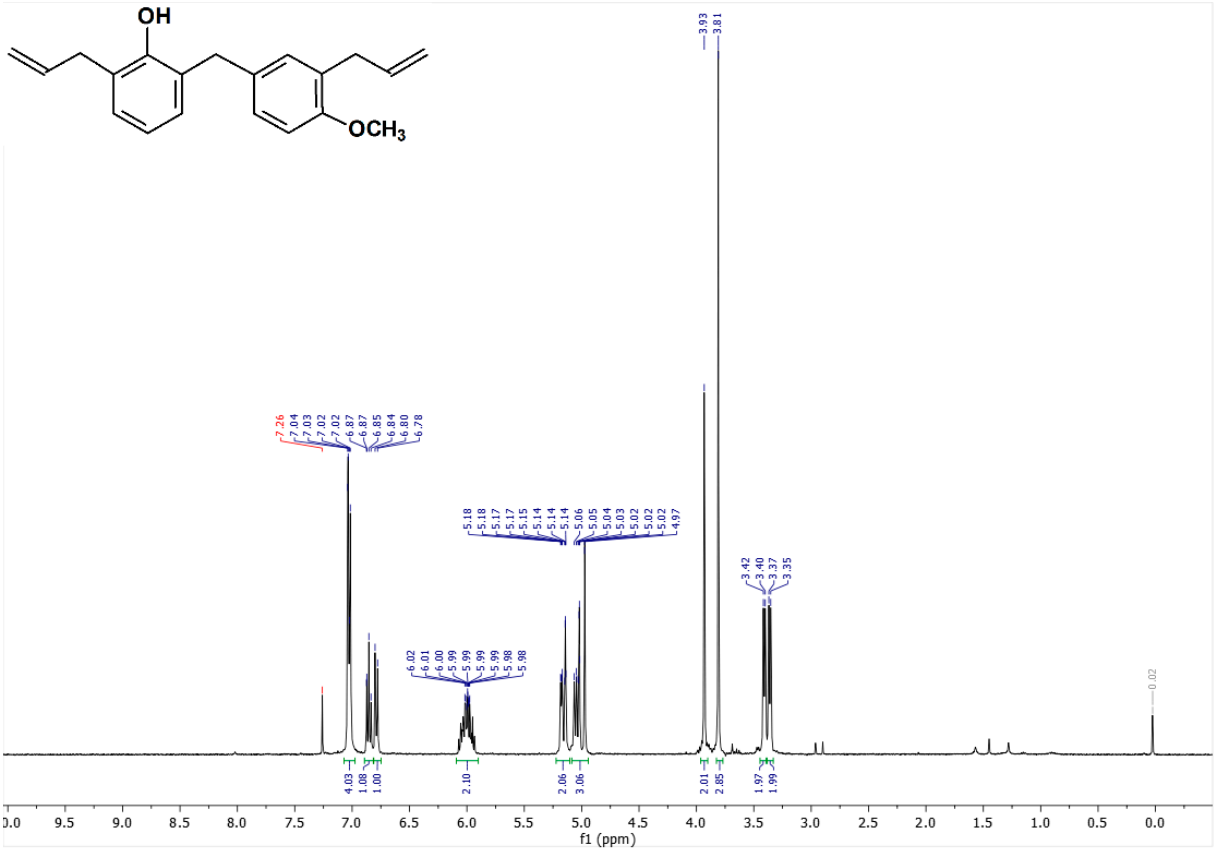

^{1}H-NMR spectrum ($CDCl_3$, 400 MHz) of **C4b**

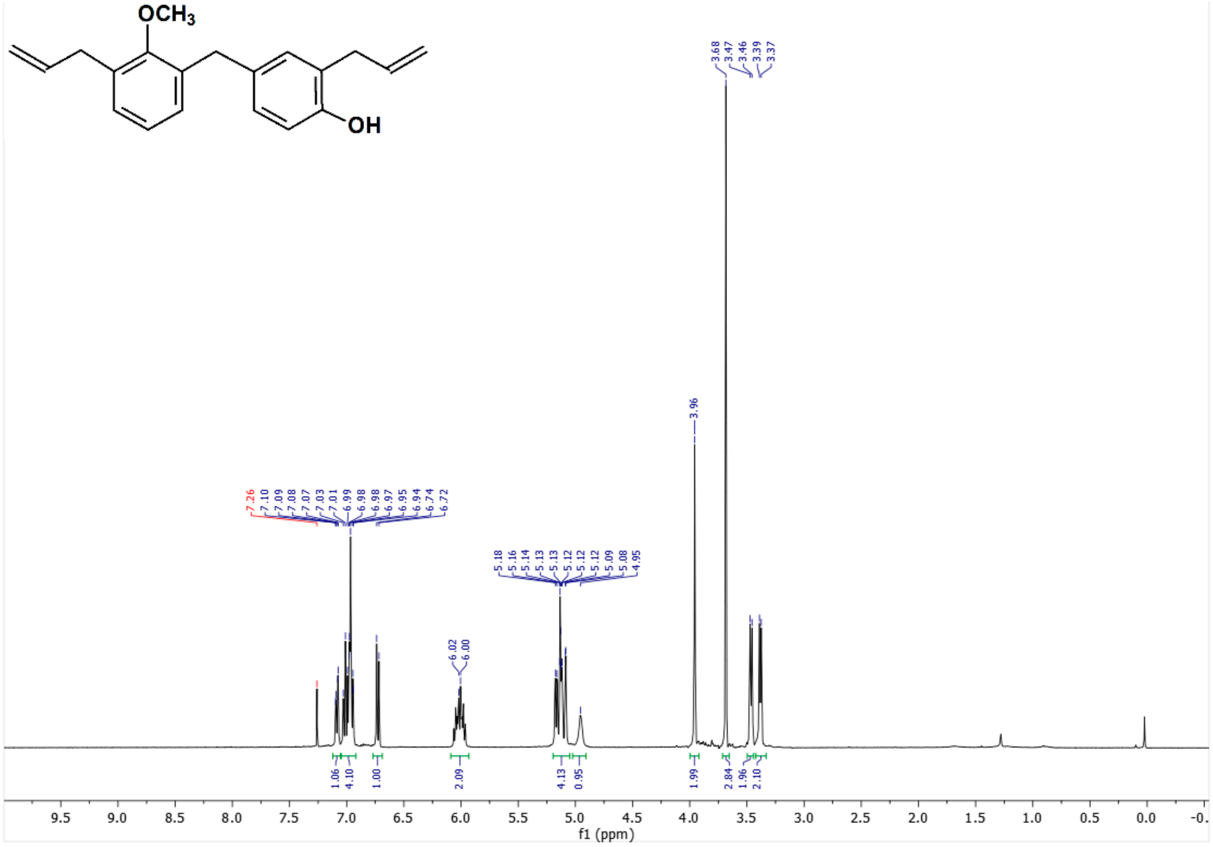

^{1}H-NMR spectrum ($CDCl_3$, 400 MHz) of **C4c**

2. Dose-Response (measured as fluorescence) curves for tested compounds

Legend to compound test codes (tables S1 to S5):

Table S1. Structures of Compounds Based on Honokiol

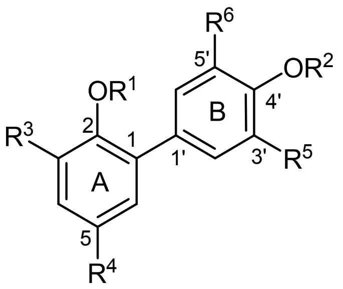

**Cpd.**	**test code**	R^1	R^2	R^3	R^4	R^5	R^6
**H**	A4	H	H	H	allyl	allyl	H
**H1**	F1	H	Me	NH_2	allyl	allyl	H
**H2**	G5	H	Me	NH-$COC_{11}H_{23}$	allyl	allyl	H
**H3**	G6	H	Me	NH-Ala	allyl	allyl	H
**H4**	G7	H	Me	NH-Phe	allyl	allyl	H
**H5**	F4	H	Et	NH_2	allyl	allyl	H
**H6**	F5	Et	Me	NH_2	allyl	allyl	H
**H7**	F6	Et	Me	NH-Ac	allyl	allyl	H
**H8**	G1	H	Me	NH-Ac	allyl	allyl	H
**H9**	G2	Me	H	H	allyl	allyl	NH-Ac
**H10**	E2	H	Et	H	allyl	allyl	H
**H11**	F7	Me	H	H	allyl	allyl	NH_2
**H12**	B5	H	H	H	allyl	1-propenyl	H
**H13**	C1	H	H	H	*n*-propyl	*n*-propyl	H
**H14**	B2	Me	H	H	allyl	allyl	H
**H15**	B1	H	Me	H	allyl	allyl	H
**H16**	B7	Me	Me	H	allyl	allyl	H
**H17**	B4	H	Me	H	allyl	1-propenyl	H
**H18**	B6	H	Me	H	2,3-dihydroxy-propyl	2,3-dihydroxy-propyl	H
**H19**	B3	H	Me	H	*n*-propyl	*n*-propyl	H
**H20**	B8	Me	Me	H	*n*-propyl	*n*-propyl	H
**H21**	E1	Et	-	H	allyl	allyl	H
**H22**	E3	H	Et	H	allyl	allyl	H
**H23**	E4	Et	H	H	*n*-propyl	*n*-propyl	H
**H24**	E5	Et	Et	H	*n*-propyl	*n*-propyl	H
**H25**	C2	H	Me	H	2-bromo-propyl	allyl	H
**H26**	E6	Me	H	H	allyl	allyl	NO_2
**H27**	E7	Me	H	H	*n*-propyl	*n*-propyl	NO_2
**H28**	E8	H	Me	NO_2	allyl	allyl	H
**H29**	F2	Me	H	H	*n*-propyl	*n*-propyl	NH_2
**H30**	F3	H	Me	NH_2	*n*-propyl	*n*-propyl	H
**H31**	G3	Me	H	H	*n*-propyl	*n*-propyl	NH-Ac
**H32**	G4	H	Me	NH-Ac	*n*-propyl	*n*-propyl	H

Table S2. Structures of Compounds Based on Biphenyl-2,2'-diol

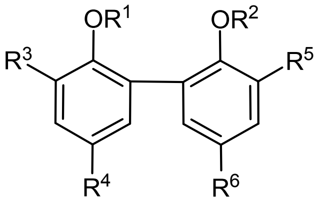

**Cpd.**	**test code**	R1	R2	R3	R4	R5	R6
**M**	A3	H	H	H	allyl	H	allyl
**M1**	A1	H	H	H	H	H	H
**M2**	A6	H	H	H	*n*-propyl	H	*n*-propyl
**M3**	A7	Me	H	H	allyl	H	allyl
**M4**	A8	Me	Me	H	allyl	H	allyl
**M5**	C5	H	H	H	Me	H	Me
**M6**	C6	H	H	H	*t*-butyl	H	*t*-butyl
**M7**	C4	H	H	allyl	H	allyl	H

Table S3. Structures of Compounds Based on Biphenyl-4,4'-diol

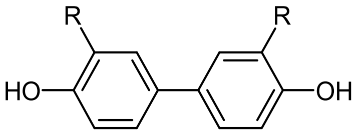

**Cpd.**	**test code**	R
**A1**	A2	H
**A2**	C5	allyl

Table S4. Structures of Compounds Based on bis(2-hydroxyphenyl)methane

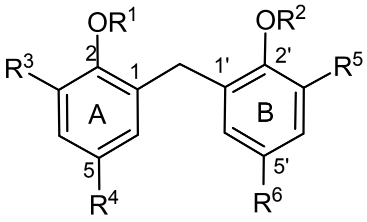

**Cpd.**	**test code**	R1	R2	R3	R4	R5	R6
**B1**	D1	H	H	H	H	H	H
**B2**	D4	H	H	allyl	H	allyl	H
**B4b**	D5	Me	H	allyl	H	allyl	H
**B4a**	D6	Me	Me	allyl	H	allyl	H

Table S5. Structures of Compounds Based on 2,4'-dihydroxydiphenylmethane

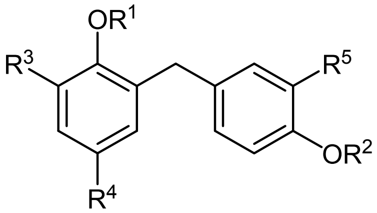

**Cpd.**	**test code**	R1	R2	R3	R4	R5
**C1**	D2	H	H	H	H	H
**C2**	D3	H	H	allyl	H	allyl
**C4c**	D8	Me	H	allyl	H	allyl
**C4b**	D7	H	Me	allyl	H	allyl
**C4a**	D9	Me	Me	allyl	H	allyl

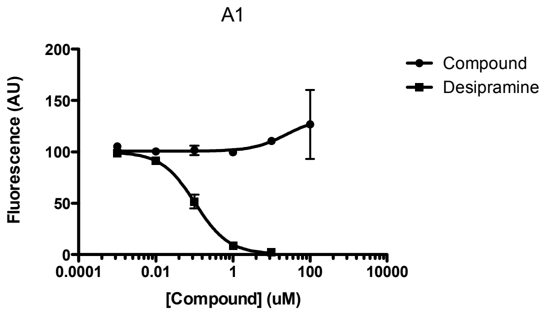

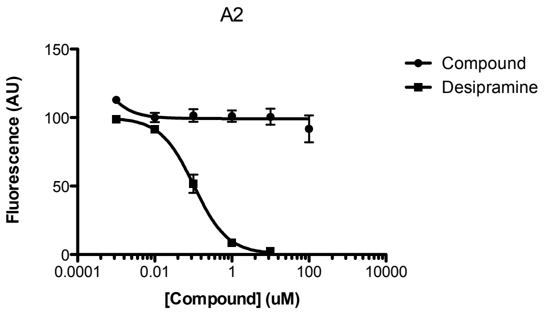

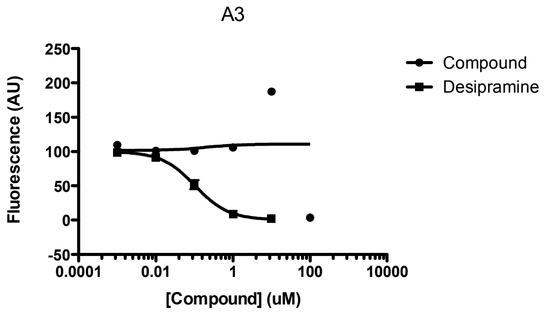

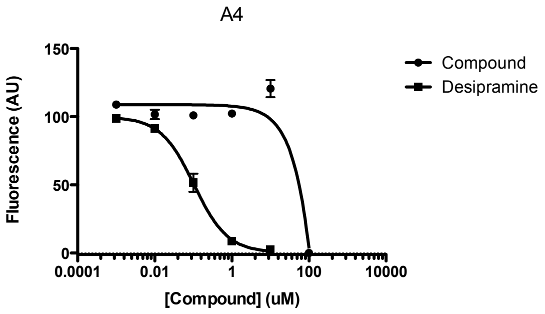

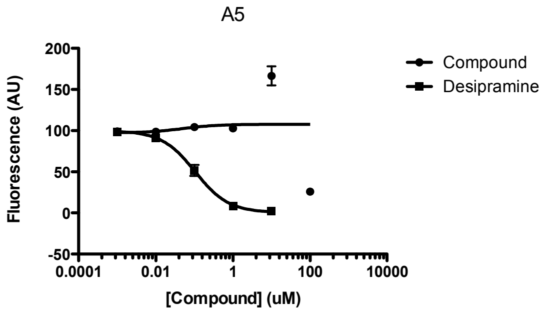

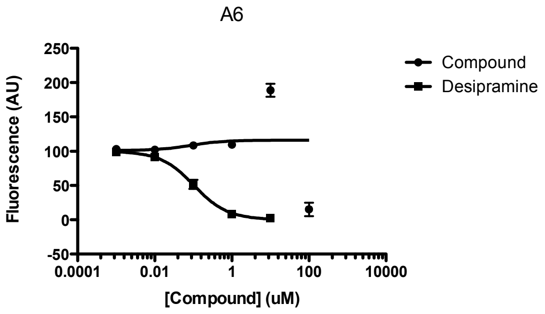

A5 does not belong to the compounds of this article

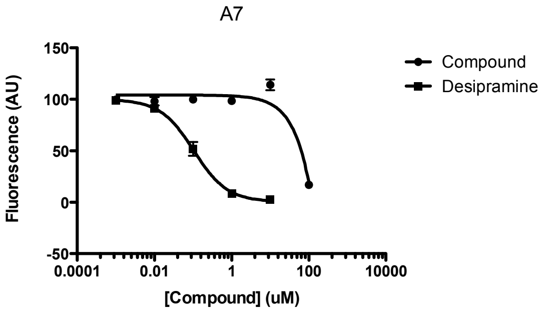
A7
Fluorescence (AU)
[Compound] (uM)
Compound
Desipramine

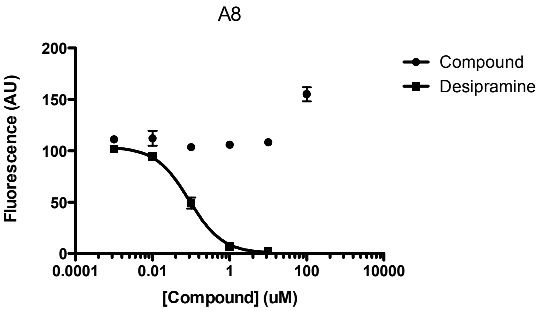
A8
Fluorescence (AU)
[Compound] (uM)
Compound
Desipramine

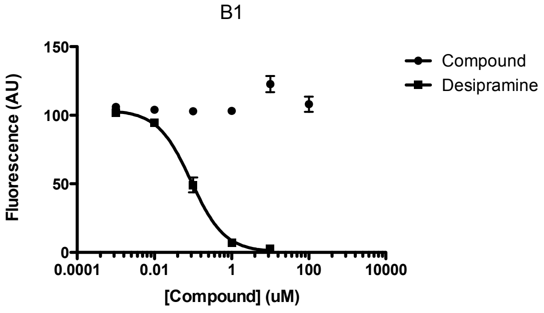
B1
Fluorescence (AU)
[Compound] (uM)
Compound
Desipramine

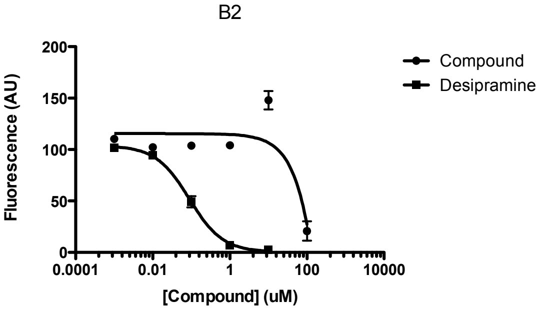
B2
Fluorescence (AU)
[Compound] (uM)
Compound
Desipramine

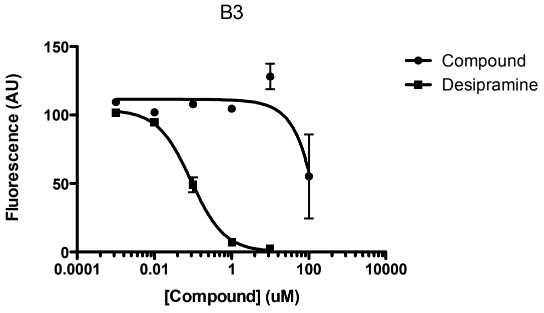
B3
Fluorescence (AU)
[Compound] (uM)
Compound
Desipramine

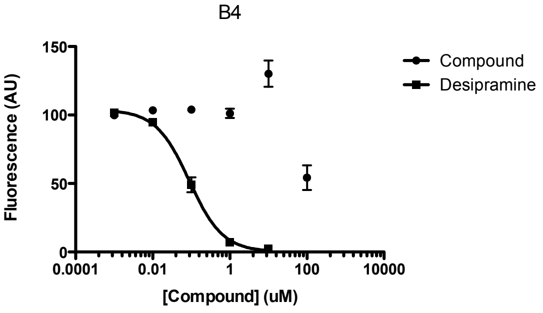
B4
Fluorescence (AU)
[Compound] (uM)
Compound
Desipramine

B5

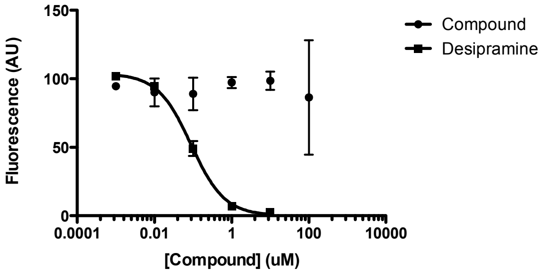

B6

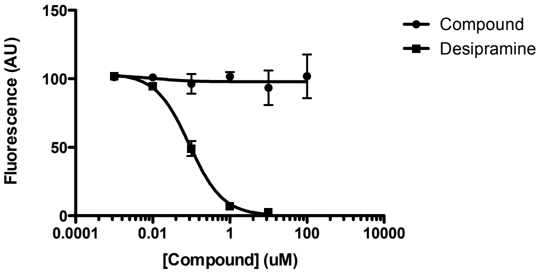

B7

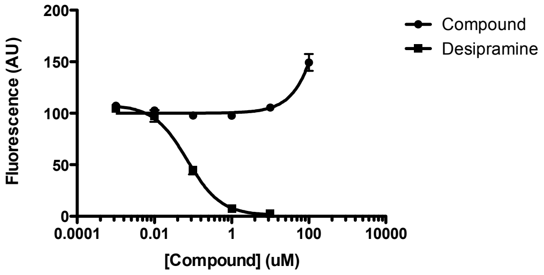

B8

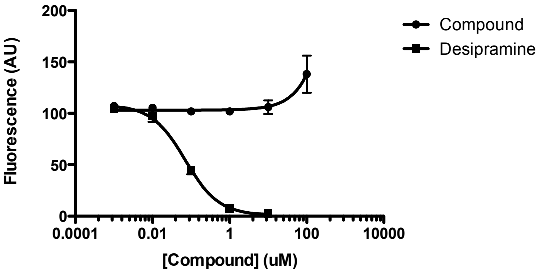

C1

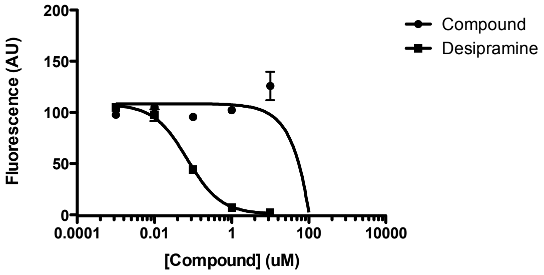

C2

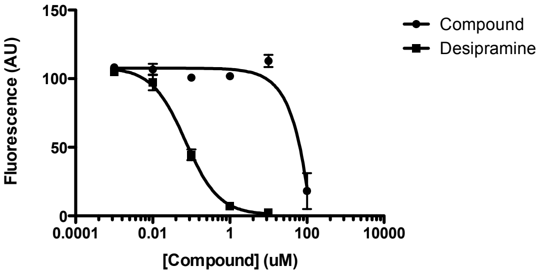

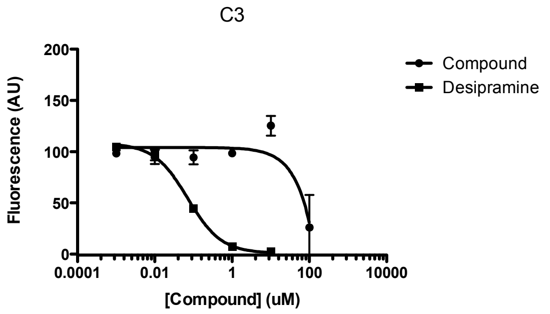

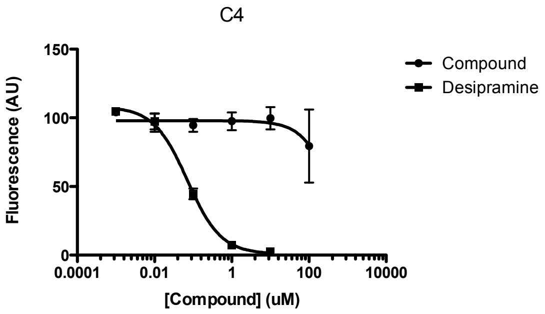

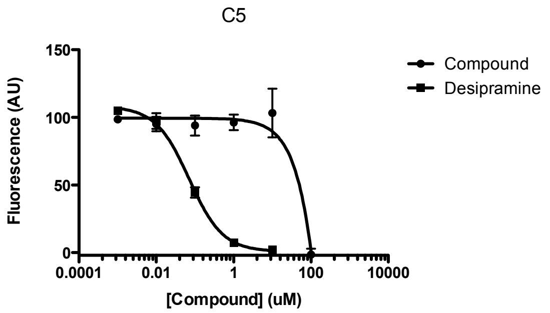

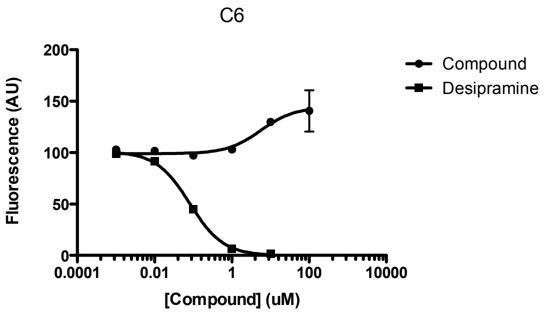

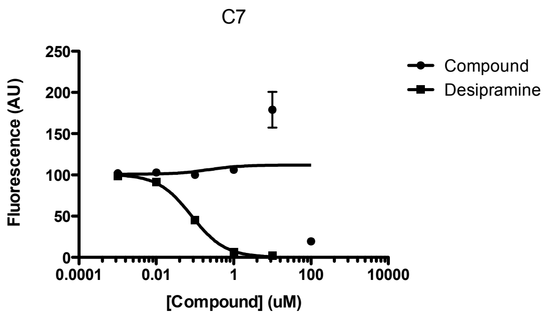

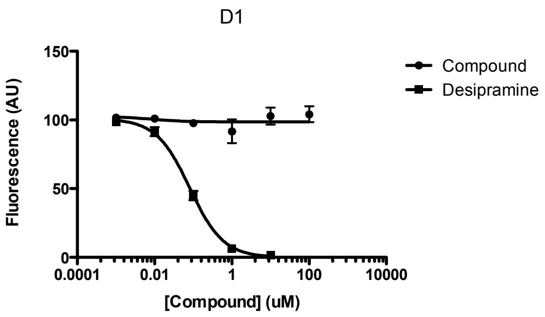

C3 does not belong to the compounds of this article

D2

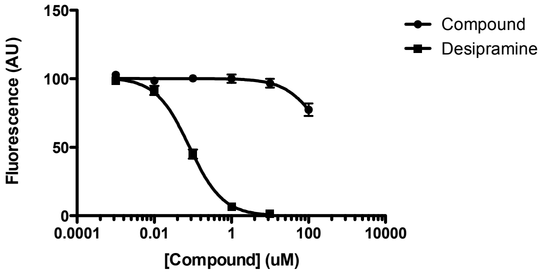

D3

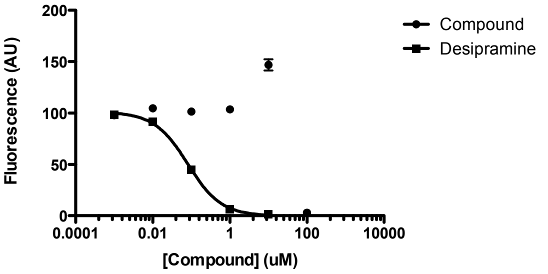

D4

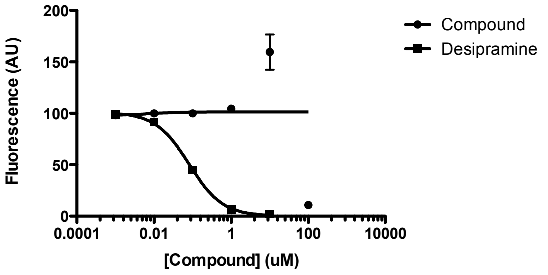

D5

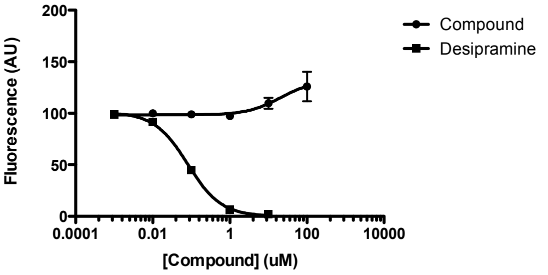

D6

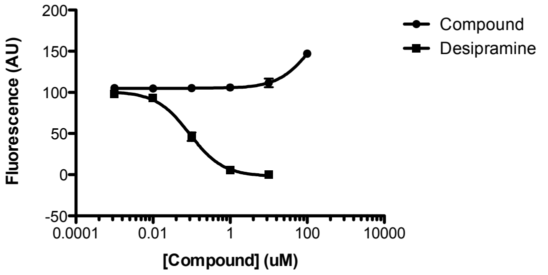

D7

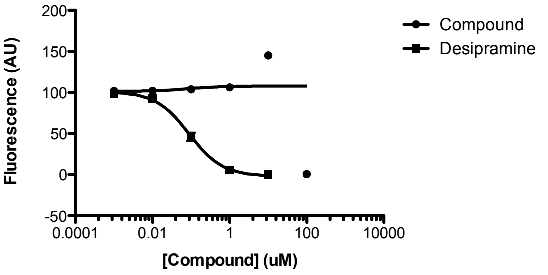

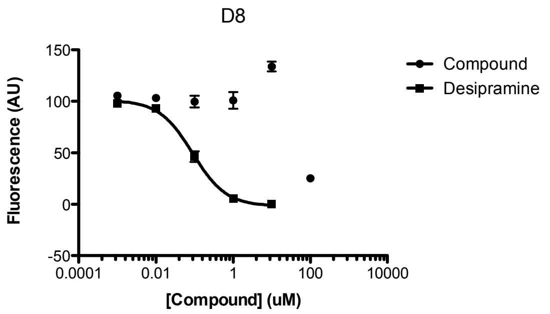
D8
Fluorescence (AU)
[Compound] (uM)
Compound
Desipramine

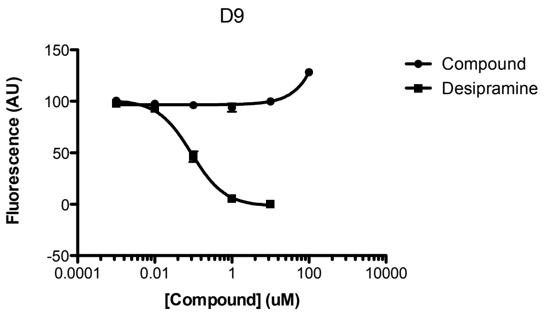
D9
Fluorescence (AU)
[Compound] (uM)
Compound
Desipramine

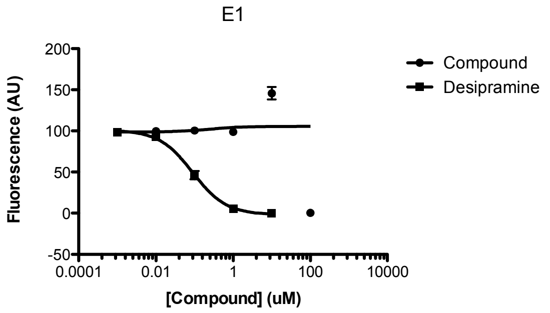
E1
Fluorescence (AU)
[Compound] (uM)
Compound
Desipramine

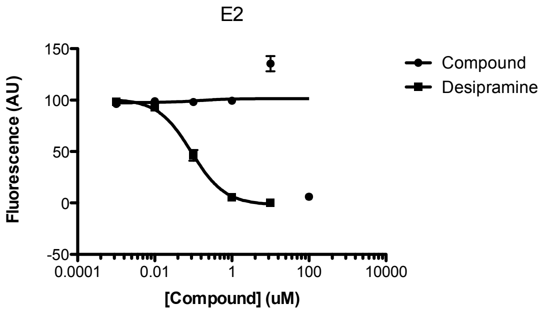
E2
Fluorescence (AU)
[Compound] (uM)
Compound
Desipramine

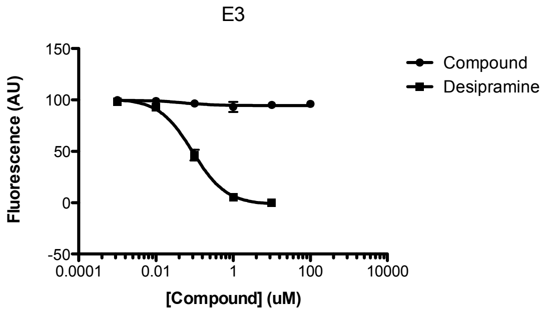
E3
Fluorescence (AU)
[Compound] (uM)
Compound
Desipramine

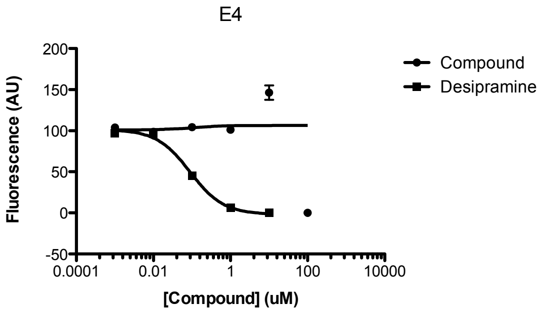
E4
Fluorescence (AU)
[Compound] (uM)
Compound
Desipramine

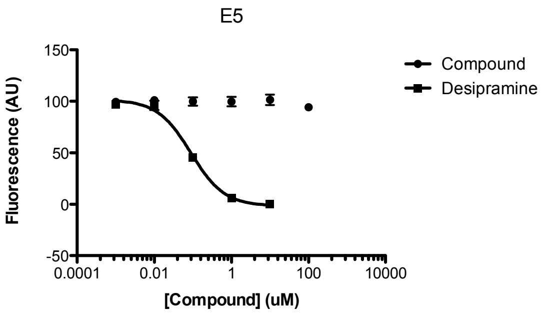
E5
Fluorescence (AU)
150
100
50
0
-50
0.0001
0.01
1
100
10000
[Compound] (uM)
Compound
Desipramine

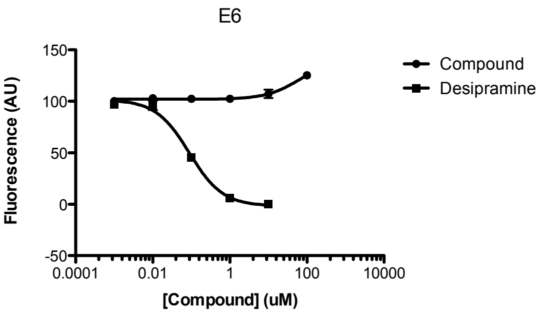
E6
Fluorescence (AU)
150
100
50
0
-50
0.0001
0.01
1
100
10000
[Compound] (uM)
Compound
Desipramine

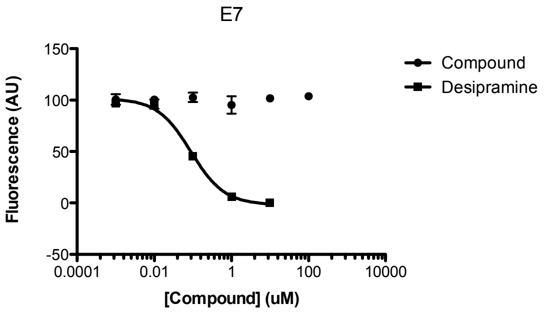
E7
Fluorescence (AU)
150
100
50
0
-50
0.0001
0.01
1
100
10000
[Compound] (uM)
Compound
Desipramine

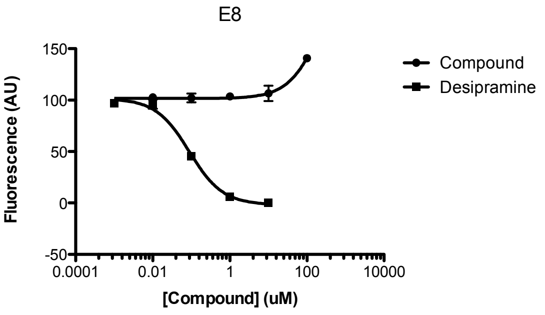
E8
Fluorescence (AU)
150
100
50
0
-50
0.0001
0.01
1
100
10000
[Compound] (uM)
Compound
Desipramine

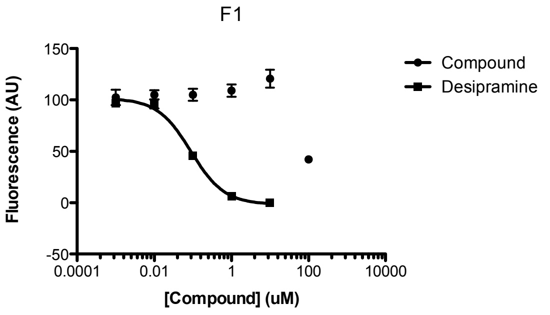
F1
Fluorescence (AU)
150
100
50
0
-50
0.0001
0.01
1
100
10000
[Compound] (uM)
Compound
Desipramine

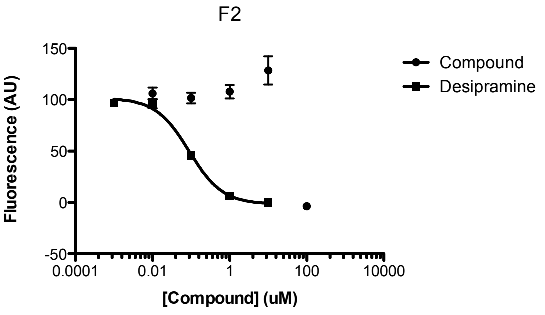
F2
Fluorescence (AU)
150
100
50
0
-50
0.0001
0.01
1
100
10000
[Compound] (uM)
Compound
Desipramine

F3

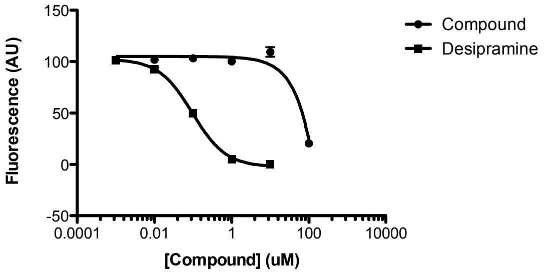

F4

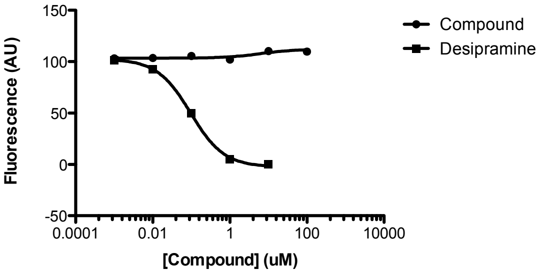

F5

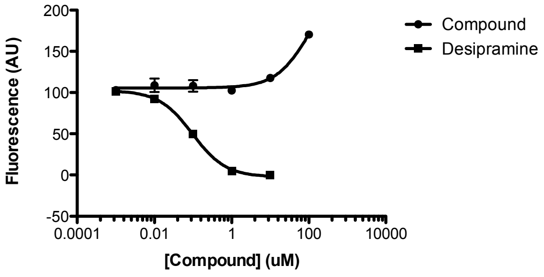

F6

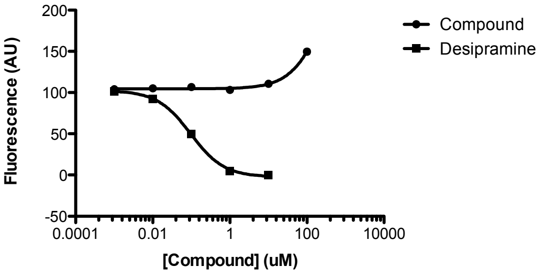

F7

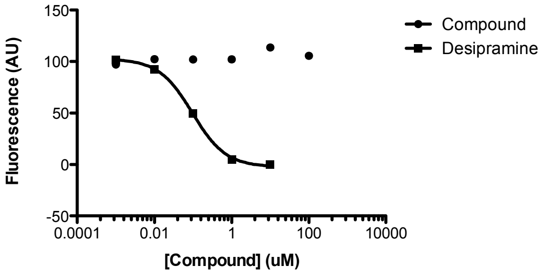

G1

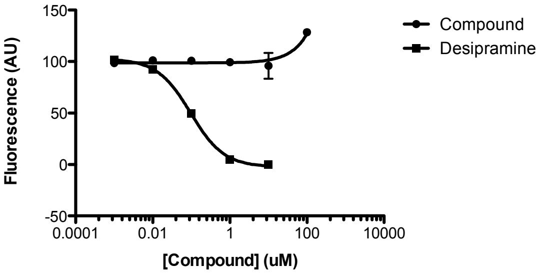

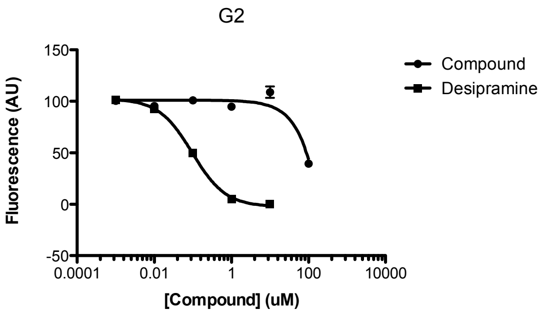
G2
Fluorescence (AU)
150
100
50
0
-50
0.0001
0.01
1
100
10000
[Compound] (uM)
Compound
Desipramine

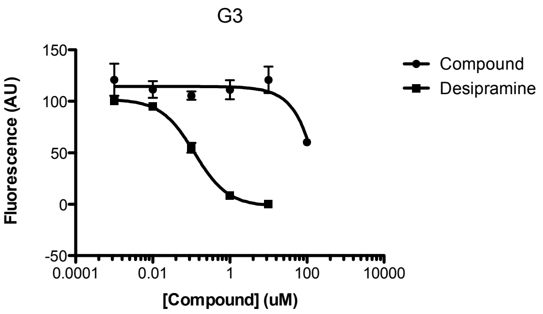
G3
Fluorescence (AU)
150
100
50
0
-50
0.0001
0.01
1
100
10000
[Compound] (uM)
Compound
Desipramine

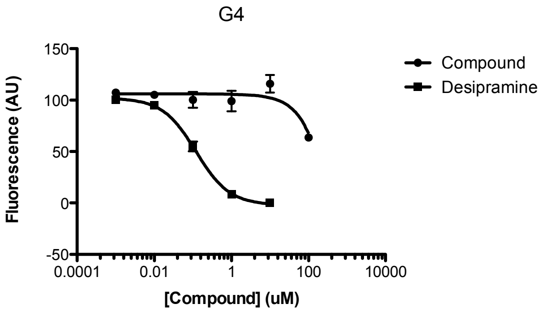
G4
Fluorescence (AU)
150
100
50
0
-50
0.0001
0.01
1
100
10000
[Compound] (uM)
Compound
Desipramine

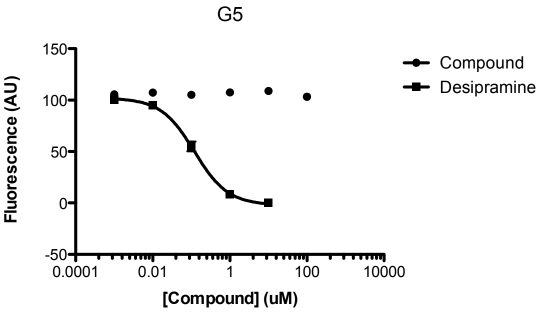
G5
Fluorescence (AU)
150
100
50
0
-50
0.0001
0.01
1
100
10000
[Compound] (uM)
Compound
Desipramine

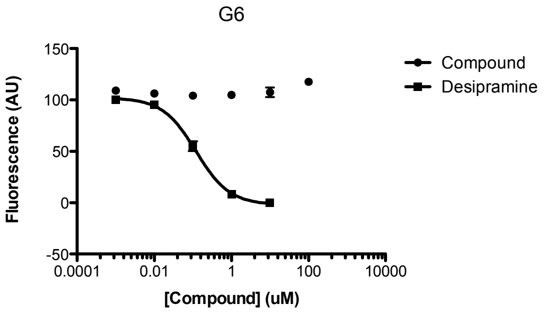
G6
Fluorescence (AU)
150
100
50
0
-50
0.0001
0.01
1
100
10000
[Compound] (uM)
Compound
Desipramine

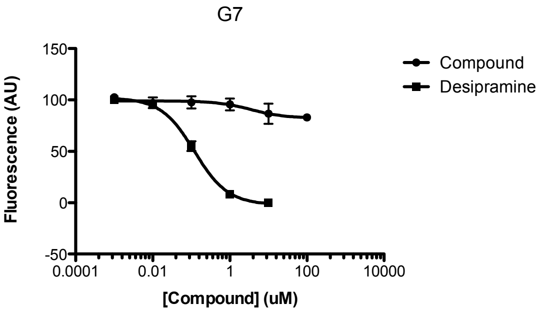
G7
Fluorescence (AU)
150
100
50
0
-50
0.0001
0.01
1
100
10000
[Compound] (uM)
Compound
Desipramine
